# Spatial-Frequency Hypergraph Neural Network for EEG-fNIRS Emotion Recognition

**DOI:** 10.3390/brainsci16090962

**Published:** 2026-09-11

**Authors:** Haifeng Li, Xueying Zhang, Guijun Chen, Yaru Zhou, Ying Sun, Lixia Huang

**Affiliations:** 1 College of Integrated Circuits, Taiyuan University of Technology, Taiyuan 030024, China; 2 College of Electronic Information Engineering, Taiyuan University of Technology, Taiyuan 030024, China

**Keywords:** emotion recognition, electroencephalogram, Functional Near-Infrared Spectroscopy, Hypergraph Neural Network, multimodal emotion recognition

## Abstract

**Background/Objectives:** Hybrid EEG-fNIRS emotion recognition aims to accurately identify an individual’s emotional state by analyzing neurophysiological signals and constitutes an important research direction in affective brain–computer interfaces and human–computer interaction. In recent years, EEG-fNIRS emotion recognition has advanced from handcrafted feature extraction and shallow fusion to deep learning and graph-based modeling. However, most existing methods rely on predefined fixed frequency-band partitioning and second-order graph structures that only support pairwise connections, making it difficult to accommodate inter-subject frequency variability and to characterize high-order brain network relationships such as multi-channel synergistic activation within a frequency band and cross-frequency coupling. **Methods:** To address these issues, this paper proposes an EEG-fNIRS emotion recognition framework based on a Spatial-Frequency Hypergraph Neural Network (SF-HGNN). First, a Dynamic Frequency Band Decomposition module is designed to achieve adaptive optimization of the EEG and fNIRS frequency bands; second, a Multi-scale Temporal Convolution module extracts temporal features at different time scales; third, a Spatial-Frequency Adaptive Hypergraph Convolution module is constructed to model intra-band cross-channel spatial synergy and channel-wise cross-frequency coupling; and finally, a Cross-Modal Attention Fusion mechanism achieves high-order interaction between the complementary information of the two modalities. The proposed method was validated on the public ENTER dataset comprising 50 participants and four emotion categories (sadness, happiness, fear, and calm). **Results:** Experimental results show that SF-HGNN achieves accuracies of 82.94% and 68.85% in subject-dependent and subject-independent experiments, respectively; ablation studies and visualization analyses further verify the effectiveness of each module and the interpretability of the model. **Conclusions:** Future work will focus on validation with larger-scale data and improving cross-subject domain generalization.

## 1. Introduction

### 1.1. Background

Emotion recognition aims to accurately identify individual emotional states by analyzing physiological or behavioral signals generated during affective processes, and constitutes an important research direction in fields such as affective brain–computer interfaces, human–computer interaction, and mental health assessment [1]. Compared with overt behavioral signals such as facial expressions and speech [2], brain signals directly reflect the underlying neural activity of the brain and are less susceptible to subjective disguise and environmental interference, thereby offering greater objectivity and reliability [3]. Electroencephalography (EEG) provides millisecond-level temporal resolution capable of capturing rapid changes in neuronal electrical activity; Functional Near-Infrared Spectroscopy (fNIRS), by contrast, offers relatively high spatial resolution that reflects cerebral hemodynamic changes in cortical regions [4,5]. These two modalities characterize emotional processing from the distinct yet complementary perspectives of neural electrical activity and cerebral hemodynamics. Hybrid EEG-fNIRS emotion recognition has therefore emerged as an important research direction in affective brain–computer interfaces.

Existing EEG-fNIRS emotion recognition methods have progressed from shallow fusion to deep cross-modal learning [6,7]. Early studies primarily adopted feature-level fusion strategies, directly concatenating handcrafted EEG temporal and frequency-domain features with fNIRS statistical features before feeding them into classifiers for emotion recognition. With the advancement of deep learning, Convolutional Neural Networks (CNNs), Transformers, and Graph Neural Networks (GNNs) have become the predominant approaches [8]. Current EEG-fNIRS research typically constructs separate feature extraction branches for EEG and fNIRS, and then combines attention mechanisms, cross-modal interaction modules, or graph structure modeling to learn complementary information between the two modalities [9,10]. These studies demonstrate that jointly modeling neural electrical activity and cerebral hemodynamic information can substantially improve emotion recognition accuracy.

Despite the significant progress achieved by existing methods, several key challenges remain. The first challenge concerns the insufficient task-adaptivity of frequency representations. The neural oscillatory rhythms and hemodynamic frequency responses associated with emotional states exhibit marked variation across different subjects, emotional tasks, and brain regions [11]. Existing studies typically partition EEG signals into canonical frequency bands—such as δ, θ, α, β, and γ [12]—and decompose fNIRS signals into frequency intervals such as very-low-frequency and low-frequency bands [13]. Although such fixed frequency band partitioning carries a degree of physiological interpretability, its assumption that all samples share identical frequency boundaries limits its ability to accommodate complex individual differences and dynamic emotional changes. This can lead to the fragmentation or aliasing of emotion-discriminative frequency components, thereby constraining the model’s capacity to represent emotion-related neural rhythms [14]. Adaptively learning the optimal frequency band boundaries of EEG and fNIRS to realize end-to-end dynamic frequency modeling thus constitutes a critical challenge in EEG-fNIRS emotion recognition.

The second challenge lies in the insufficient modeling of high-order interactions within brain networks. Emotional processing arises not from the isolated activity of a single brain region or electrode channel, but from the synergistic coordination of multiple electrode channels across several cortical regions under different neural rhythms [15]. Graph Neural Networks have been widely applied to EEG and fNIRS brain network modeling, learning spatial topological features across brain regions or channels via connectivity relationships among electrodes. Conventional graph structures, however, permit an edge to connect only two nodes, inherently describing only second-order relationships between nodes [16]. When applied to multi-band, multi-channel EEG-fNIRS signals, this limitation manifests in two ways. First, emotion-related brain activity within a single frequency band typically involves the synchronized activation of multiple channels across different brain regions, yet pairwise connections cannot model the entire set of nodes as a unified whole. Second, cross-frequency coupling is pervasive among distinct neural rhythms within the same channel, and relying solely on pairwise connections cannot adequately capture the complex associations that exist along the frequency dimension. Consequently, traditional second-order graph structures alone are insufficient to fully characterize group-level synergistic activity and high-order spatial-frequency interactions within brain networks.

Furthermore, EEG and fNIRS exhibit pronounced differences in sampling rates and spatiotemporal resolutions, and the manner in which emotional information is expressed across modalities is highly heterogeneous [17]. Most existing fusion methods, however, rely on shallow integration—such as feature concatenation, weighted summation, or simple attention mechanisms. Lacking targeted, fine-grained semantic alignment, they fail to enable effective interaction among the core complementary information across modalities. Overcoming modal heterogeneity and establishing precise cross-modal semantic interaction mechanisms to unlock the full synergistic potential of EEG and fNIRS thus constitutes another pressing challenge for current emotion recognition research.

### 1.2. Related Work

#### 1.2.1. EEG-fNIRS Emotion Recognition

Early studies primarily relied on handcrafted features extracted from EEG or fNIRS signals for emotion recognition [18]. For EEG signals, commonly used features include power spectral density, differential entropy, differential asymmetry, and Hjorth parameters; for fNIRS signals, researchers mainly focused on concentration changes in oxygenated hemoglobin (HbO) and deoxygenated hemoglobin (HbR) together with their statistical properties such as integral values, slopes, and peak amplitudes [19,20]. These handcrafted features were subsequently fed into traditional machine learning classifiers such as Support Vector Machines (SVMs) to perform emotional state discrimination. Such methods depend on prior domain knowledge to design features, making it difficult to fully extract the deep discriminative information embedded in the signals; moreover, the feature extraction and classification stages are decoupled, limiting the model’s capacity for end-to-end optimization.

With the advancement of deep learning, CNNs and Transformers have been introduced into end-to-end feature learning for EEG and fNIRS. By exploiting local receptive fields, CNNs can effectively capture spatial and frequency-domain patterns in signals. For example, EEGNet [21] extracts EEG features via temporal convolutions and depthwise separable convolutions, achieving compact and efficient EEG representation learning. For EEG and fNIRS modality fusion, relevant studies typically construct separate feature extraction branches for EEG and fNIRS and then accomplish cross-modal information interaction through feature concatenation, weighted summation, or attention mechanisms. For instance, MFA-CNN [8] employs CNNs to extract fNIRS temporal features and EEG spatial-frequency features, respectively, and fuses information from both modalities via a modality-frequency attention mechanism. Such methods validate the effectiveness of jointly modeling neural electrical activity and cerebral hemodynamic information for enhancing emotion recognition performance. Nevertheless, because EEG and fNIRS differ substantially in sampling frequencies and spatiotemporal resolutions, and because emotional information is expressed in highly heterogeneous forms across the two modalities, existing fusion methods remain confined to shallow feature interaction and cannot fully exploit the advantages of cross-modal synergistic complementarity. Additionally, CNNs and Transformers inherently model signals using regular grids or sequential structures [22] and cannot explicitly capture the complex non-Euclidean topological relationships among brain regions—a limitation that has motivated researchers to introduce graph structures into brain network modeling.

#### 1.2.2. Brain Network Topological Structure Modeling

To explicitly model topological interactions among brain regions, GNNs have been introduced into EEG- and fNIRS-based emotion recognition. By treating electrode channels or brain regions as graph nodes and edges as inter-node relationships, GNNs can learn topological representations in non-Euclidean space that align with the organizational properties of the brain [23]. Early methods typically constructed static adjacency matrices based on spatial electrode distances, statistical correlations, or functional connectivity, and leveraged Graph Convolutional Networks (GCNs) for node feature aggregation [24]. For instance, RGNN constructed functional brain connectivity graphs to capture inter-electrode interactions, validating the efficacy of graph-based representations in emotion recognition [25]. Static graphs, however, struggle to adapt to the dynamic brain network patterns that vary across subjects and emotional states. To enhance adaptability, researchers subsequently incorporated Graph Attention Networks (GATs) [26] along with dynamic graph learning techniques—such as DGCNN [27] and ST-DGAT [28]—to capture individualized, state-dependent functional connectivity patterns by learning adaptive inter-node weights or dynamically generating graph structures conditioned on the input samples.

Although the aforementioned methods learn topological representations between EEG and fNIRS channels, they are constrained to pairwise second-order connections between nodes: an edge can only connect two nodes, making it impossible to model the synchronized activation of multiple brain-region channels within the same frequency band as an integrated whole, and rendering it difficult to represent multi-dimensional joint relationships such as cross-frequency coupling within a single channel [29]. To transcend this limitation, Hypergraph Neural Networks (HGNNs) have been introduced into brain signal analysis. Unlike edges in ordinary graphs that can connect only two nodes, hyperedges in hypergraphs can simultaneously connect an arbitrary number of nodes, thereby representing high-order group relationships among multiple brain regions in a more natural manner [30]. For example, to address the difficulty that traditional GNNs face in describing multi-regional synergistic activity, STHGCN [15] proposed a spatiotemporal hypergraph convolutional network that employs spatial hypergraphs to model multi-channel high-order brain-region associations and temporal hypergraphs to capture dynamic dependencies in EEG sequences, thereby extending the modeling capability from second-order connections to high-order interactions. Hyper-MML [31] further extended hypergraph learning to multimodal emotion recognition, modeling high-order interactions among EEG, audio, and video by constructing intra-segment and inter-segment hyperedges to achieve effective multimodal information fusion.

### 1.3. Main Contributions

To address the above challenges, we propose an EEG-fNIRS emotion recognition framework based on a Spatial-Frequency Hypergraph Neural Network (SF-HGNN). First, a Dynamic Frequency Band Decomposition (DyFreBD) module is designed to achieve adaptive optimization of EEG and fNIRS frequency representations via learnable frequency band boundaries. Subsequently, a Multi-scale Temporal Convolution (MTC) module is employed to extract multi-scale temporal features, and a Spatial-Frequency Adaptive Hypergraph Convolution (SF-AHGC) module is constructed to respectively model intra-band cross-channel spatial synergies and channel-wise cross-frequency coupling relationships. Finally, a Cross-Modal Attention Fusion (CMAF) mechanism achieves deep interaction between EEG and fNIRS features, yielding a more discriminative joint brain representation. The main contributions of this work are as follows:

- We propose an EEG-fNIRS emotion recognition framework based on SF-HGNN. By unifying dynamic frequency learning, high-order spatial-frequency relationship modeling, and Cross-Modal Attention Fusion, the framework achieves end-to-end joint learning of complementary information between EEG and fNIRS.
- We propose the DyFreBD module, which parameterizes frequency band boundaries as learnable variables and adaptively optimizes the frequency partitioning of EEG and fNIRS through a differentiable filtering mechanism, thereby overcoming the limited adaptability of fixed frequency band strategies to individual differences and task variations.
- We propose the SF-AHGC module, which captures cross-channel synergies and cross-frequency coupling relationships using spatial hypergraphs and frequency hypergraphs, respectively, and achieves adaptive modeling of high-order brain network structures via dynamic association weights.
- We systematically evaluate the proposed framework on a public EEG-fNIRS emotion dataset through subject-dependent and subject-independent experiments, assessing its effectiveness from the perspectives of recognition performance and interpretability.

### 1.4. Research Objectives and Research Questions

To address the above challenges, the objective of this study is to construct an EEG-fNIRS emotion recognition framework that integrates dynamic frequency learning, high-order spatial-frequency relationship modeling, and Cross-Modal Attention Fusion, enabling end-to-end joint modeling of the complementary information between the two modalities. Based on this objective, we formulate the following research questions (RQs):

RQ1: Can treating frequency band boundaries as learnable parameters enable the model to obtain frequency band partitions that adapt to individual differences?

RQ2: Do the DyFreBD, MTC, SF-AHGC, and CMAF modules all contribute positively to recognition performance?

RQ3: Can the spatial and frequency hypergraphs learn structured association patterns that reflect intra-band cross-channel synergy and channel-wise cross-frequency coupling?

The remainder of this paper is organized as follows. Section 2 describes the EEG-fNIRS emotion dataset used in this study and details the proposed SF-HGNN model. Section 3 presents the experimental setup and results, encompassing the dataset, evaluation protocols, subject-dependent experiments, subject-independent experiments, and ablation analyses. Section 4 discusses the experimental findings in relation to the state of the art. Section 5 concludes the paper and outlines directions for future work.

## 2. Materials and Methods

### 2.1. Participants

The experiments were conducted on the publicly available ENTER dataset [32], which comprises simultaneous EEG and fNIRS recordings for emotion recognition from 50 college students, including 25 males (age: 22.92 ± 1.71 years) and 25 females (age: 24.12 ± 1.67 years). All participants were right-handed, had normal or corrected-to-normal vision and normal hearing, and had no history of mental illness or drug use. Written informed consent was obtained from all participants prior to the experiments. The study protocol was approved by the Institutional Review Board of Taiyuan University of Technology and complied with the guidelines of the Declaration of Helsinki.

### 2.2. Instruments

**Figure 1:**
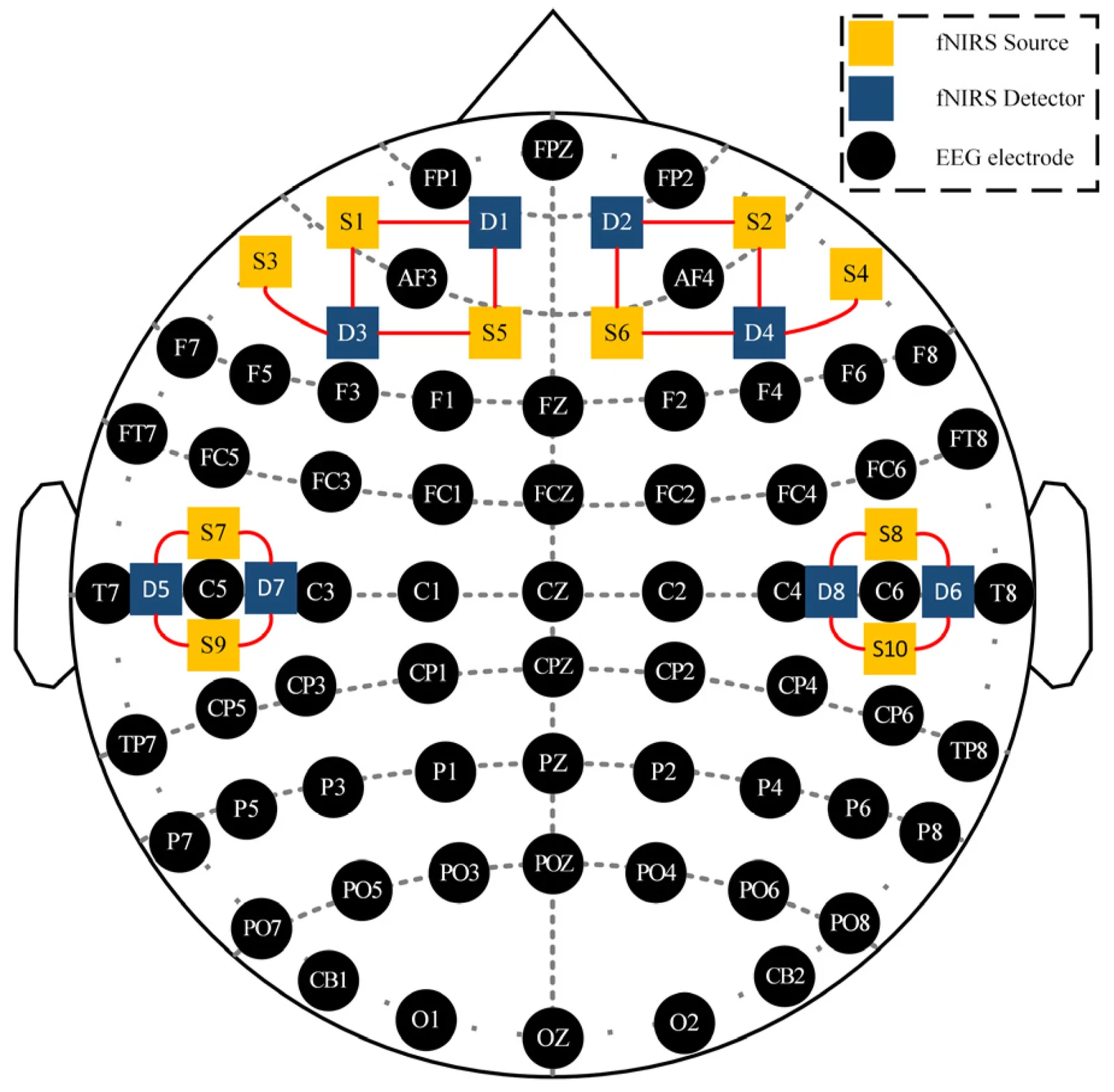
Spatial configuration of EEG electrodes, fNIRS light sources, and detectors.

EEG signals were recorded using a Neuroscan SynAmps2 system (Neuroscan USA, Ltd., Charlotte, NC, USA) with 64 electrode channels at a sampling rate of 1000 Hz, with electrodes arranged according to the extended international 10–20 system; fNIRS signals were recorded using a NirSmart device (DanYang HuiChuang Medical Equipment Co., Ltd., Danyang, China) with 18 optode channels at a sampling rate of 11 Hz. The EEG electrodes and fNIRS optodes were placed on the same cap without physical contact, ensuring synchronized recording of the two modalities; their spatial configuration is shown in Figure 1.

The emotion-eliciting materials consisted of 60 pre-selected video clips organized into four emotion categories—sadness, happiness, fear, and calm—with 15 clips per category and each clip lasting 1–2 min. Before the formal experiment, 20 non-participating raters evaluated the clips along the valence and arousal dimensions using the Self-Assessment Manikin (SAM) to ensure that the selected materials could reliably elicit the target emotions. All experiments were conducted in an electromagnetically shielded room.

### 2.3. Procedure

**Figure 2:**
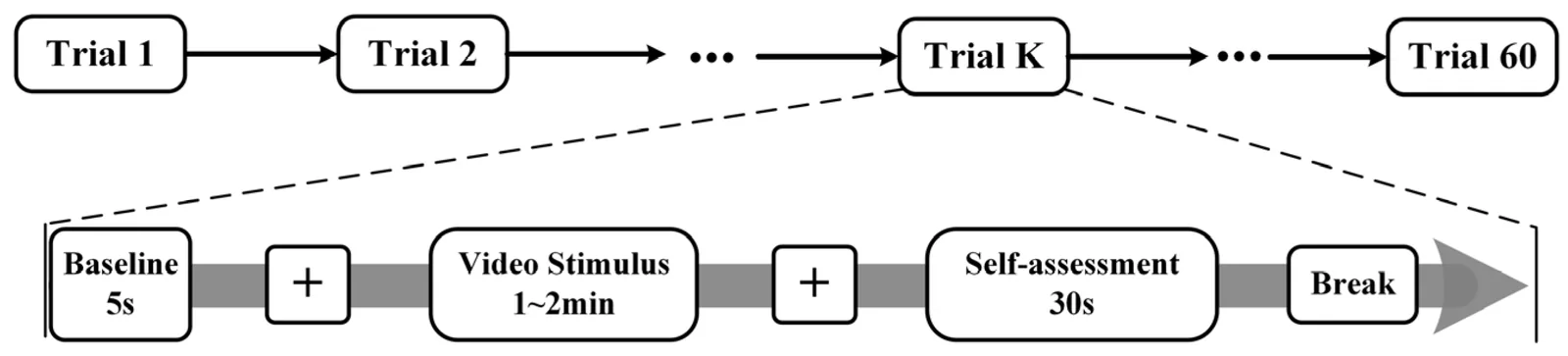
Flowchart of the ENTER dataset collection procedure.

The experimental procedure is illustrated in Figure 2. Before the formal experiment, the experimenter explained the experimental procedure and precautions to each participant in detail. Each trial consisted of three phases: a 5 s resting preparation, followed by watching one emotion-eliciting video clip (1–2 min), and then completing an evaluation form within 30 s to judge the emotion type elicited by the video, after which the next trial began. Each participant completed 60 trials in total, with 15 trials for each of the four emotion categories. During the experiment, participants sat in a chair facing a computer screen and were asked to keep their head still and avoid large movements as much as possible to reduce motion artifacts.

### 2.4. Preprocessing

Raw EEG signals are primarily affected by ocular artifacts, electromyographic artifacts, power-line frequency interference, and electrode drift [33]. To address these artifact sources, we applied average re-referencing, baseline correction, band-pass filtering (0.1–75 Hz), notch filtering (50 Hz), per-channel Z-score normalization (with clipping at the 0.5th and 99.5th percentiles), and downsampling to 200 Hz.

Raw fNIRS optical density signals are primarily affected by physiological noise—including cardiac pulsation, respiration, and vasomotor oscillations—as well as low-frequency instrumental drift [5]. To address these noise sources, we first performed baseline correction and band-pass filtering (0.01–0.2 Hz). The filtered optical density signals were then converted to $\Delta c_{HbO}$ and $\Delta c_{HbR}$ concentration changes using the modified Beer–Lambert law [34]. Finally, per-channel Z-score normalization was applied. The modified Beer–Lambert law is given by Equation (1):

(1) $$\begin{array}{cc} A & =\ln\left(\frac{I_{0}}{I}\right)\\ & ={\mathrm{DPF}}^{\lambda }\cdot \sum_{i=1}^{n}\varepsilon_{i}^{\lambda }\cdot c_{i}\cdot l\\ & ={\mathrm{DPF}}^{\lambda }\cdot l\cdot \left(\varepsilon_{HbO}^{\lambda }\cdot c_{HbO}+\varepsilon_{HbR}^{\lambda }\cdot c_{HbR}\right) \end{array}$$

where A denotes the absorbance, $I_{0}$ is the incident light intensity, I is the detected light intensity, $\varepsilon^{\lambda }$ is the molar extinction coefficient of the chromophore, c is the chromophore concentration, and l is the optical pathlength—i.e., the distance between the emission optode and the detection optode of the fNIRS system. *DPF* denotes the differential pathlength factor, a dimensionless correction factor that accounts for the increased optical pathlength caused by the scattering properties of biological tissue. By solving the system of equations in Equation (2) using absorbance values at different time points and wavelengths, the concentration changes $\Delta c_{HbO}$ and $\Delta c_{HbR}$ are obtained.

(2) $$\left(\begin{array}{c} \Delta c_{HbO}\\ \Delta c_{HbR} \end{array}\right)={\left(\begin{array}{cc} \varepsilon_{HbO}^{\lambda_{1}} & \varepsilon_{HbR}^{\lambda_{1}}\\ \varepsilon_{HbO}^{\lambda_{2}} & \varepsilon_{HbR}^{\lambda_{2}}\\ \varepsilon_{HbO}^{\lambda_{3}} & \varepsilon_{HbR}^{\lambda_{3}} \end{array}\right)}^{-1}\cdot \left(\begin{array}{c} \frac{\Delta A^{\lambda_{1}}}{l\cdot DPF^{\lambda_{1}}}\\ \frac{\Delta A^{\lambda_{2}}}{l\cdot DPF^{\lambda_{2}}}\\ \frac{\Delta A^{\lambda_{3}}}{l\cdot DPF^{\lambda_{3}}} \end{array}\right)$$

A temporally synchronized sliding window approach was adopted to segment the dual-modal data. The sliding stride for both modalities was uniformly fixed at 1 s to ensure temporal alignment. For EEG, the window length was set to 1 s, corresponding to a sample length of 200 time points. For fNIRS, the window length was set to 18.18 s, likewise corresponding to a sample length of 200 time points. After segmentation, EEG and fNIRS samples sharing the same timestamp were paired to form dual-modal samples each containing 200 time points, with their corresponding emotion labels recorded synchronously.

### 2.5. Spatial-Frequency Hypergraph Neural Network

**Figure 3:**
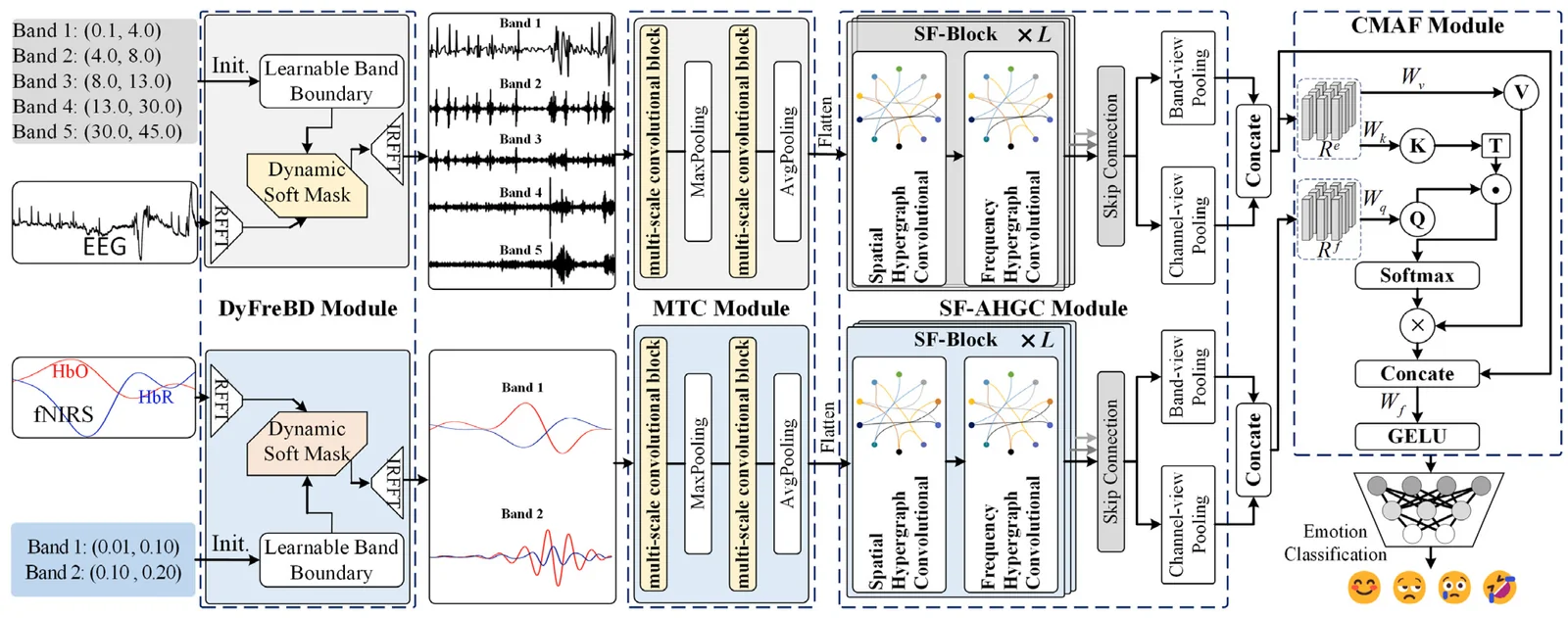
Overall architecture of the Spatial-Frequency Hypergraph Neural Network.

We propose an EEG-fNIRS emotion recognition framework built on a Spatial-Frequency Hypergraph Neural Network (SF-HGNN). The framework accepts EEG and fNIRS as input and models the spatiotemporal-frequency features of each modality through two structurally symmetric, parametrically independent feature extraction sub-networks. As illustrated in Figure 3, SF-HGNN comprises a Dynamic Frequency Band Decomposition module, a Multi-scale Temporal Convolution module, a Spatial-Frequency Adaptive Hypergraph Convolution module, and a Cross-Modal Attention Fusion module. DyFreBD learns the optimal frequency band boundaries for EEG and fNIRS in an end-to-end differentiable manner; MTC employs parallel asymmetric convolution kernels to capture local temporal patterns at multiple receptive fields; SF-AHGC models cross-channel spatial synergy within frequency bands via spatial hypergraph convolution and cross-band coupling within individual channels via frequency hypergraph convolution, thereby capturing high-order interactions in EEG and fNIRS signals; and CMAF fuses the two modalities through a cross-modal attention mechanism, yielding emotionally discriminative EEG-fNIRS joint representations.

The EEG and fNIRS signals, together with their initial frequency band boundaries, serve as model inputs. The EEG input tensor has shape $x^{e}\in {\mathbb{R}}^{C_{e}\times T_{e}}$, where $C_{e}$ and $T_{e}$ denote the channel count and the number of time points, respectively. The fNIRS input tensor has shape $x^{f}\in {\mathbb{R}}^{C_{f}\times T_{f}}$, where the channel count $C_{f}$ is formed by concatenating the oxyhemoglobin subset $X^{o}$ and the deoxyhemoglobin subset $X^{r}$, and $T_{f}$ is the number of fNIRS time points. Both signals are processed by the DyFreBD module and decomposed into $K_{e}$ and $K_{f}$ frequency bands, producing multi-band feature tensors $X^{e}\in {\mathbb{R}}^{N\times K_{e}\times C_{e}\times T_{e}}$ and $X^{f}\in {\mathbb{R}}^{N\times K_{f}\times C_{f}\times T_{f}}$, where N denotes the total number of samples. Through cross-validation, the data are partitioned into a training set $D_{tr}=\{X_{tr}^{e},X_{tr}^{f},Y_{tr}^{emo}\}$ and a test set $D_{te}=\{X_{te}^{e},X_{te}^{f},Y_{te}^{emo}\}$, with $Y^{emo}\in {\mathbb{Z}}^{N}$ representing the emotion labels.

#### 2.5.1. Dynamic Frequency Band Decomposition

Different frequency bands of EEG and fNIRS correspond to distinct neural oscillatory rhythms and hemodynamic response patterns. Yet, the frequency bands sensitive to a given emotional task differ substantially both across individuals and between modalities. Methods that rely on pre-defined, fixed frequency band boundaries cannot produce optimal frequency-domain representations for a given subject and task. We therefore introduce the DyFreBD module, which treats frequency band boundaries as trainable parameters optimized during end-to-end training so that the model adaptively discovers the frequency bands most informative for emotion recognition.

For a single-modality input signal $x\in {\mathbb{R}}^{C\times T}$ with effective frequency range $\left[f_{\min},f_{\max}\right]$ and a target of K frequency bands, DyFreBD introduces K−1 learnable internal boundary parameters ${\left\{r_{k}\right\}}_{k=1}^{K-1}$. These are constrained to the normalized interval (0,1) by a Sigmoid function and then mapped to the actual frequency range:

(3) $$b_{k}=f_{\min}+\sigma (r_{k})\cdot (f_{\max}-f_{\min}),\;\;\;\;k=1,\ldots ,K-1$$

All boundaries are sorted and fixed endpoints $b_{0}=f_{\min}$ and $b_{K}=f_{\max}$ are prepended and appended, yielding K contiguous, non-overlapping frequency band intervals ${\left\{[b_{k-1},b_{k}]\right\}}_{k=1}^{K}$. Because the spectral characteristics of EEG and fNIRS differ markedly, DyFreBD implements independent Dynamic Frequency Band Decomposition mechanisms for the two modalities.

(a). EEG Dynamic Frequency Band Decomposition

**Figure 4:**
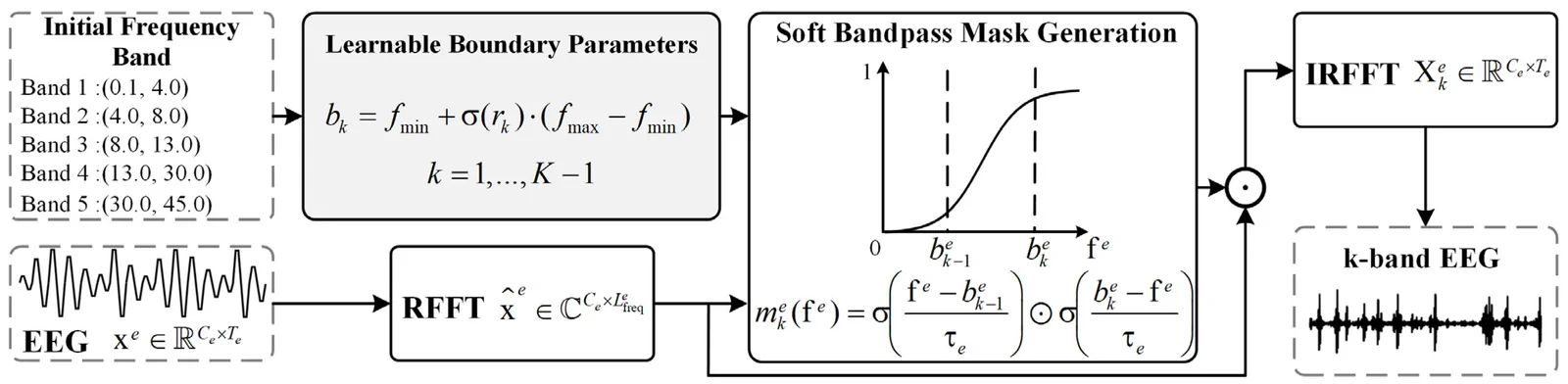
Architecture of the EEG Dynamic Frequency Band Decomposition.

As shown in Figure 4, DyFreBD first applies a Real Fast Fourier Transform (RFFT) of length $T_{e}$ along the temporal dimension of the EEG signal $x^{e}\in {\mathbb{R}}^{C_{e}\times T_{e}}$ to obtain the single-sided complex spectrum:

(4) $${\hat{x}}^{e}=\mathrm{RFFT}(x^{e},T_{e})\in {\mathbb{C}}^{C_{e}\times L_{\mathrm{freq}}^{e}}$$

where the number of frequency bins is $L_{\mathrm{freq}}^{e}=\left\lfloor T_{e}/2\right\rfloor +1$ and the corresponding discrete frequency vector is $f^{e}=[0,\;f_{s}^{e}/T_{e},\;\ldots ,\;f_{s}^{e}/2]$. For the k-th frequency band, the band-pass soft mask is defined as the element-wise product of two Sigmoid functions:

(5) $$m_{k}^{e}(f^{e})=\sigma \;\left(\frac{f^{e}-b_{k-1}^{e}}{\tau_{e}}\right)⊙\sigma \;\left(\frac{b_{k}^{e}-f^{e}}{\tau_{e}}\right)$$

where $\tau_{e}$ is a hyperparameter that controls the roll-off steepness of the EEG filter transition band. After element-wise multiplication of the soft mask with the complex spectrum, an Inverse Real Fast Fourier Transform (IRFFT) recovers the time-domain sub-signal of the k-th EEG frequency band:

(6) $$X_{k}^{e}=\mathrm{IRFFT}\;\left({\hat{x}}^{e}⊙m_{k}^{e}(f^{e}),T_{e}\right)\in {\mathbb{R}}^{C_{e}\times T_{e}}$$

Repeating this operation for all $K_{e}$ frequency bands and concatenating along the frequency band dimension produces the multi-band EEG feature tensor $X^{e}\in {\mathbb{R}}^{K_{e}\times C_{e}\times T_{e}}$.

(b). fNIRS Dynamic Frequency Band Decomposition

Compared with EEG, fNIRS has a far lower sampling rate, and its effective frequency content is confined to the extremely low frequency range. Under a standard discretization, gradient points within the transition band are sparse, impeding effective boundary parameter updates. DyFreBD therefore extends the transform length of the fNIRS signal to $N_{\mathrm{pad}}^{f}$ via zero-padding, raising the frequency resolution in the low-frequency region to $f_{s}^{f}/N_{\mathrm{pad}}^{f}$ and supplying a sufficiently dense gradient computation path for the soft mask boundaries:

(7) $${\hat{x}}^{f}=\mathrm{RFFT}(x^{f},N_{\mathrm{pad}}^{f})\in {\mathbb{C}}^{C_{f}\times L_{\mathrm{freq}}^{f}},\;\;\;\;f^{f}=\left[0,\;\frac{f_{s}^{f}}{N_{\mathrm{pad}}^{f}},\;\ldots ,\;\frac{f_{s}^{f}}{2}\right]$$

where $L_{\mathrm{freq}}^{f}=\left\lfloor N_{\mathrm{pad}}^{f}/2\right\rfloor +1$. For the k-th fNIRS frequency band, the soft mask is constructed as:

(8) $$m_{k}^{f}(f^{f})=\sigma \;\left(\frac{f^{f}-b_{k-1}^{f}}{\tau_{f}}\right)⊙\sigma \;\left(\frac{b_{k}^{f}-f^{f}}{\tau_{f}}\right)$$

where $\tau_{f}$ is a temperature hyperparameter controlling the roll-off steepness of the fNIRS filter transition band. During backpropagation, DyFreBD injects a custom gradient amplification mechanism into the fNIRS branch. While leaving the forward computation unchanged, it selectively boosts the gradient sensitivity of boundary parameters in the extremely low-frequency transition band. This compensates for the weak parameter updates that would otherwise result from sparse gradients in this frequency range. The optimized soft mask is multiplied with the spectrum, and an IRFFT with $N_{\mathrm{pad}}^{f}$ padding points transforms the result back to the original $T_{f}$ time steps:

(9) $$X_{k}^{f}=\mathrm{IRFFT}\;{\left({\hat{x}}^{f}⊙m_{k}^{f}(f^{f}),N_{\mathrm{pad}}^{f}\right)}_{\left[\ldots ,:T_{f}\right]}\in {\mathbb{R}}^{C_{f}\times T_{f}}$$

Repeating this operation for all $K_{f}$ frequency bands and concatenating along the frequency band dimension yields the multi-band fNIRS feature tensor $X^{f}\in {\mathbb{R}}^{K_{f}\times C_{f}\times T_{f}}$.

#### 2.5.2. Multi-Scale Temporal Convolution

After decomposition by DyFreBD, each frequency band sub-signal of EEG and fNIRS carries both short-range local transient patterns and long-range global rhythmic patterns in the time domain. Both types of temporal structure contribute materially to accurate emotion decoding. To capture temporal emotional features across multiple time scales, we construct parametrically independent MTC feature extractors for the two modalities.

(a). EEG Multi-scale Temporal Convolution

**Figure 5:**
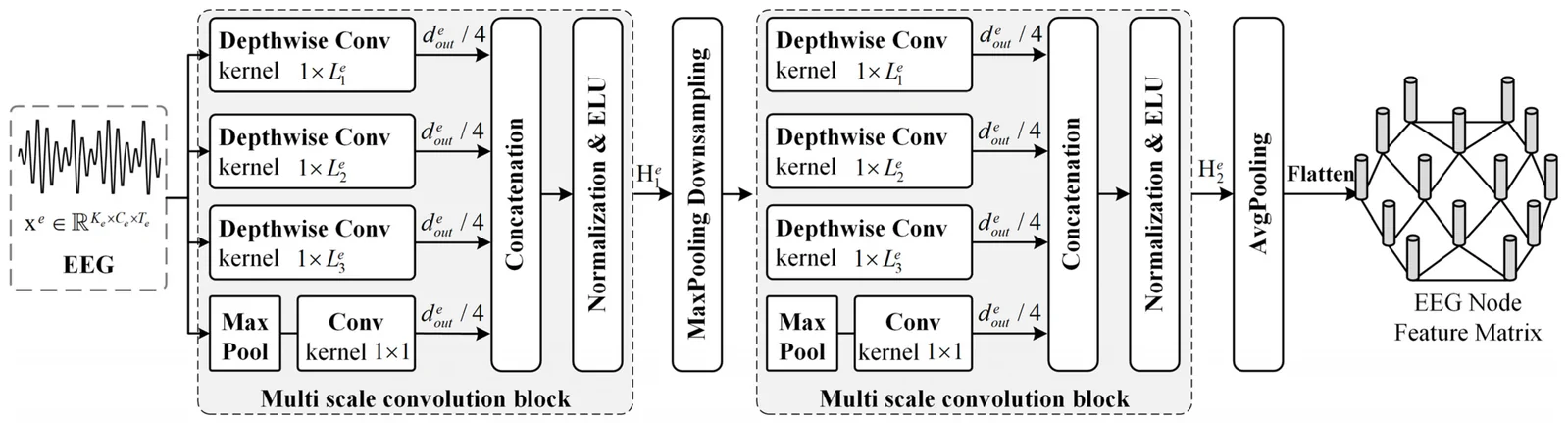
Network architecture of the EEG Multi-scale Temporal Convolution.

The EEG Multi-scale Temporal Convolution module first expands the EEG multi-band tensor $X^{e}\in {\mathbb{R}}^{N\times K_{e}\times C_{e}\times T_{e}}$ along the frequency band dimension and reshapes it into ${\tilde{X}}^{e}\in {\mathbb{R}}^{(N\cdot K_{e})\times 1\times C_{e}\times T_{e}}$, so that each frequency band undergoes temporal modeling independently within the merged sample space. As shown in Figure 5, the module consists of two cascaded convolution blocks, each containing four parallel temporal feature extraction branches. To preserve the spatial topology of EEG, all branches employ depthwise convolution, operating solely along the temporal dimension while leaving channel dimensions independent.

The first three convolution branches adopt temporal convolution kernels of $L_{1}^{e}$, $L_{2}^{e}$, and $L_{3}^{e}$ at distinct scales to capture both transient fluctuations and long-range trends in neural signals:

(10) $$Y_{m}^{e}=C_{L_{m}^{e}}({\tilde{X}}^{e}),\;\;\;\;m\in \{1,2,3\}$$

where $C_{L_{m}^{e}}(\cdot )$ denotes a temporal convolution operator with kernel size $1\times L_{m}^{e}$. The fourth branch extracts locally salient activation features via temporal max pooling, followed by a 1×1 convolution for channel dimension alignment:

(11) $$Y_{4}^{e}=C_{1}\;\left(P_{L_{p}^{e}}({\tilde{X}}^{e})\right)$$

where $P_{L_{p}^{e}}(\cdot )$ denotes a temporal max pooling operator with kernel size $1\times L_{p}^{e}$ and stride 1, and $C_{1}(\cdot )$ is a 1×1 channel convolution. The four branch outputs are concatenated along the channel dimension and passed through a Batch Normalization (BN) layer followed by an Exponential Linear Unit (ELU) activation, yielding the output $H_{1}^{e}$ of the first multi-scale convolution block:

(12) $$H_{1}^{e}=\mathrm{ELU}\;\left(\mathrm{BN}\;\left(\left[Y_{1}^{e};\;Y_{2}^{e};\;Y_{3}^{e};\;Y_{4}^{e}\right]\right)\right)\in {\mathbb{R}}^{(N\cdot K_{e})\times d_{out}^{e}\times C_{e}\times T_{1}^{e}}$$

where [⋅] denotes channel-wise concatenation. Each branch outputs $d_{out}^{e}/4$ channels, yielding $d_{out}^{e}$ channels after concatenation, and $T_{1}^{e}$ is the temporal dimension length retained after padding. Next, $H_{1}^{e}$ is temporally downsampled by a max pooling layer with kernel size and stride both equal to $1\times L_{ds}^{e}$:

(13) $${\tilde{H}}_{1}^{e}=P_{L_{ds}^{e}}(H_{1}^{e})\in {\mathbb{R}}^{(N\cdot K_{e})\times d_{out}^{e}\times C_{e}\times T_{2}^{e}}$$

where $T_{2}^{e}=\left\lfloor T_{1}^{e}/L_{ds}^{e}\right\rfloor$. The second multi-scale convolution block performs the identical four-branch extraction and fusion on the downsampled features ${\tilde{H}}_{1}^{e}$:

(14) $$H_{2}^{e}=\mathrm{ELU}\;\left(\mathrm{BN}\;\left(\left[C_{L_{1}^{e}}({\tilde{H}}_{1}^{e});\;C_{L_{2}^{e}}({\tilde{H}}_{1}^{e});\;C_{L_{3}^{e}}({\tilde{H}}_{1}^{e});\;C_{1}\;\left(P_{L_{p}^{e}}({\tilde{H}}_{1}^{e})\right)\right]\right)\right)\in {\mathbb{R}}^{(N\cdot K_{e})\times d\times C_{e}\times T_{2}^{e}}$$

After the second multi-scale convolution block, adaptive average pooling collapses the temporal dimension to 1. Dimension permutation and flattening then produce the EEG node feature matrix $Z^{e}$ of shape $N\times N_{e}\times d$, where $N_{e}=K_{e}\times C_{e}$ is the total number of frequency-channel joint nodes and d is the hidden feature dimension.

(b). fNIRS Multi-scale Temporal Convolution

The fNIRS Multi-scale Temporal Convolution module analogously expands the fNIRS multi-band tensor $X^{f}\in {\mathbb{R}}^{N\times K_{f}\times C_{f}\times T_{f}}$ along the frequency band dimension and reshapes it into ${\tilde{X}}^{f}\in {\mathbb{R}}^{(N\cdot K_{f})\times 1\times C_{f}\times T_{f}}$. Given that fNIRS signals primarily reflect low-frequency hemodynamic responses whose temporal rate of change is significantly slower than that of EEG, this branch likewise adopts depthwise convolution but is configured with parametrically independent parallel kernels with larger receptive fields. The feature extraction and fusion of the first multi-scale convolution block proceed as:

(15) $$H_{1}^{f}=\mathrm{ELU}\;\left(\mathrm{BN}\;\left(\left[C_{L_{1}^{f}}({\tilde{X}}^{f});\;C_{L_{2}^{f}}({\tilde{X}}^{f});\;C_{L_{3}^{f}}({\tilde{X}}^{f});\;C_{1}\;\left(P_{L_{p}^{f}}({\tilde{X}}^{f})\right)\right]\right)\right)\in {\mathbb{R}}^{(N\cdot K_{f})\times d_{out}^{f}\times C_{f}\times T_{1}^{f}}$$

where $C_{L_{m}^{f}}(\cdot )\;(m=1,2,3)$ denotes a temporal convolution operator with kernel size $1\times L_{m}^{f}$; the hyperparameters $\left\{L_{1}^{f},L_{2}^{f},L_{3}^{f}\right\}$ set the temporal receptive field of each branch to accommodate slowly evolving hemodynamic trends. $P_{L_{p}^{f}}(\cdot )$ is a temporal max pooling operator (kernel size $1\times L_{p}^{f}$, stride 1) that captures locally salient hemodynamic activations, and $C_{1}(\cdot )$ performs dimension alignment. Each branch outputs $d_{out}^{f}/4$ channels, giving $d_{out}^{f}$ channels after concatenation. A second convolution block is then cascaded, with a max pooling layer (kernel size and stride $1\times L_{ds}^{f}$) inserted between the blocks for temporal downsampling:

(16) $${\tilde{H}}_{1}^{f}=P_{L_{ds}^{f}}(H_{1}^{f})\in {\mathbb{R}}^{(N\cdot K_{f})\times d_{out}^{f}\times C_{f}\times T_{2}^{f}}$$

(17) $$H_{2}^{f}=\mathrm{ELU}\;\left(\mathrm{BN}\;\left(\left[C_{L_{1}^{f}}({\tilde{H}}_{1}^{f});\;C_{L_{2}^{f}}({\tilde{H}}_{1}^{f});\;C_{L_{3}^{f}}({\tilde{H}}_{1}^{f});\;C_{1}\;\left(P_{L_{p}^{f}}({\tilde{H}}_{1}^{f})\right)\right]\right)\right)\in {\mathbb{R}}^{(N\cdot K_{f})\times d\times C_{f}\times T_{2}^{f}}$$

where $T_{2}^{f}=\left\lfloor T_{1}^{f}/L_{ds}^{f}\right\rfloor$. After the second multi-scale convolution block, adaptive average pooling removes the temporal dimension, and dimension permutation and flattening produce the fNIRS node feature matrix $Z^{f}$ of shape $N\times N_{f}\times d$, where $N_{f}=K_{f}\times C_{f}$ is the total number of fNIRS frequency-channel joint nodes.

#### 2.5.3. Spatial-Frequency Adaptive Hypergraph Convolution

Emotional states arise from the synergistic coupling of multiple functional brain regions across both spatial and frequency dimensions, fundamentally reflecting high-order collective interactions within neural populations [35]. Conventional Graph Neural Networks, however, only support pairwise second-order connections between nodes, which imposes two limitations when modeling the joint spatial-frequency features of EEG and fNIRS. First, the emotional response across multiple channels within a single frequency band constitutes a set-level spatial synergy: pairwise edges decompose this collective behavior into numerous isolated dyadic connections rather than modeling the channel group as an integrated whole. Second, cross-frequency coupling exists among different bands within the same channel, yet pairwise edges are equally incapable of explicitly representing the high-order joint state of a single channel spanning all frequency bands. We therefore propose the SF-AHGC module, which exploits the defining property of hyperedges—the ability to connect an arbitrary number of nodes simultaneously—to construct separate spatial and frequency hypergraphs that model the high-order collective interactions of EEG and fNIRS along the spatial and frequency dimensions, respectively.

(a). Spatial-Frequency Adaptive Association Matrix

Let m∈{e,f} denote the modality, where e indexes EEG and f indexes fNIRS. The spatial-frequency hypergraph is $G^{m}=(V^{m},E^{m})$. The node set $V^{m}$ contains $N_{m}=K_{m}\times C_{m}$ frequency-channel joint nodes; each node $v_{\left(k,c\right)}^{m}$ corresponds to the feature representation of the c-th channel within the k-th frequency band. The hyperedge set $E^{m}$ comprises two types of high-order connections grounded in explicit neurophysiological priors, with $E_{m}=K_{m}+C_{m}$ hyperedges in total.

- Spatial hyperedges, totaling $K_{m}$, group all channel nodes within a single frequency band into one high-order set to model spatial synergy among channels in that band. The k-th spatial hyperedge $e_{k}^{s,m}$ connects all $C_{m}$ channel nodes within the frequency band k:
  (18) $$e_{k}^{s,m}=\left\{v_{\left(k,c\right)}^{m}|c=1,\ldots ,C_{m}\right\}$$
- Frequency hyperedges, totaling $C_{m}$, group all frequency-band nodes within a single channel into one high-order set to model the nonlinear cross-band coupling of that channel. The c-th frequency hyperedge $e_{c}^{f,m}$ connects the nodes spanning all $K_{m}$ frequency bands within channel c:
  (19) $$e_{c}^{f,m}=\left\{v_{\left(k,c\right)}^{m}|k=1,\ldots ,K_{m}\right\}$$

The topological structures of these two hyperedge types are fixed across samples and thus cannot capture the dynamic reorganization of brain network topology under different emotional states. To address this, we decompose the association matrix into the product of a fixed structural mask and a learnable soft weight, preserving the neurophysiological prior constraints while introducing a sample-adaptive mechanism that modulates connectivity strength. The fixed structural masks $M^{s,m}\in {\left\{0,1\right\}}^{N_{m}\times K_{m}}$ and $M^{f,m}\in {\left\{0,1\right\}}^{N_{m}\times C_{m}}$ encode the node-to-hyperedge membership of the spatial and frequency hypergraphs, respectively; non-zero entries correspond to the physical membership relationships defined above.

Given the current-layer input node features $Z^{m}\in {\mathbb{R}}^{N\times N_{m}\times d}$, the soft membership strengths of nodes to spatial hyperedges are generated dynamically via a learnable projection matrix $W_{mem}^{s,m}\in {\mathbb{R}}^{d\times K_{m}}$ and a Sigmoid activation:

(20) $$S^{s,m}=\sigma \;\left(Z^{m}W_{mem}^{s,m}\right)\in {\mathbb{R}}^{N\times N_{m}\times K_{m}}$$

Fusing this soft membership matrix with the structural mask via the Hadamard product yields the dynamic spatial association matrix:

(21) $$H_{d}^{s,m}=S^{s,m}⊙M^{s,m}\in {\mathbb{R}}^{N\times N_{m}\times K_{m}}$$

where the non-zero entries of $H_{d}^{s,m}$ are continuous values in (0,1) that represent the dynamic membership strength of a node to a given spatial hyperedge, while the zero entries are strictly enforced by $M^{s,m}$, preserving the original physical topological boundaries. Analogously, the soft membership strengths of nodes to frequency hyperedges are generated via a learnable projection matrix $W_{mem}^{f,m}\in {\mathbb{R}}^{d\times C_{m}}$, producing the dynamic frequency association matrix:

(22) $$S^{f,m}=\sigma \;\left(Z^{m}W_{mem}^{f,m}\right)\in {\mathbb{R}}^{N\times N_{m}\times C_{m}},\;\;\;\;H_{d}^{f,m}=S^{f,m}⊙M^{f,m}\in {\mathbb{R}}^{N\times N_{m}\times C_{m}}$$

**Figure 6:**
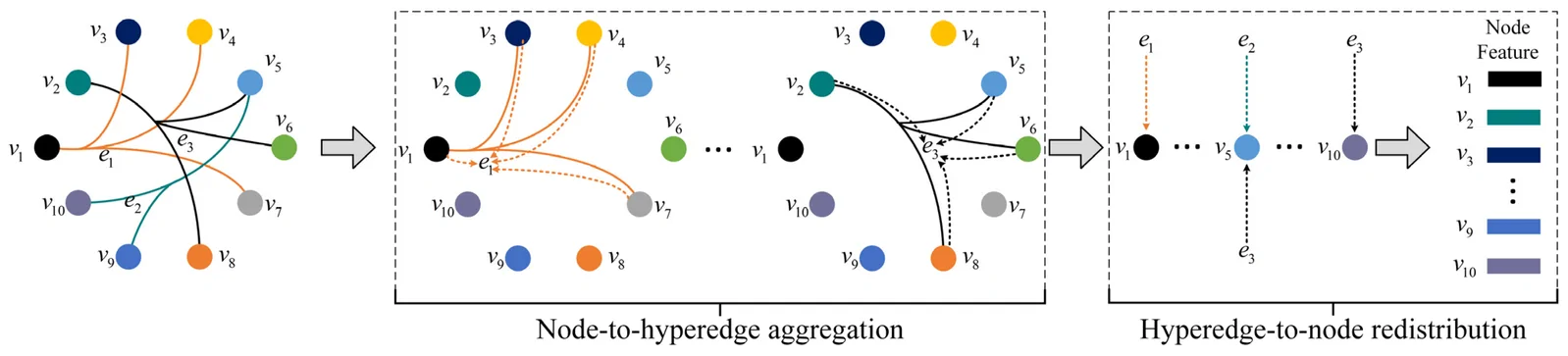
Two-stage training procedure of SF-AHGC.

SF-AHGC then performs node-to-hyperedge aggregation and hyperedge-to-node redistribution alternately within the spatial hypergraph convolution and the frequency hypergraph convolution, thereby learning high-order spatial synergy within each frequency band and high-order frequency coupling within each channel. Figure 6 illustrates this two-stage training procedure.

(b). Spatial Hypergraph Convolution

Spatial hypergraph convolution takes node features $Z^{m}\in {\mathbb{R}}^{N\times N_{m}\times d}$ as input and aggregates high-order cross-channel spatial synergy within each frequency band through two alternating stages.

- **Node-to-hyperedge aggregation.** Given the dynamic spatial association matrix $H_{d}^{s,m}$ and the dynamic hyperedge degree matrix $D_{e}^{s,m}\in {\mathbb{R}}^{N\times K_{m}\times K_{m}}$, degree-normalized hypergraph convolution is performed:
  (23) $$E^{s,m}={\left(D_{e}^{s,m}\right)}^{-1}{\left(H_{d}^{s,m}\right)}^{⊤}Z^{m}\in {\mathbb{R}}^{N\times K_{m}\times d}$$
  The diagonal entries of $D_{e}^{s,m}$ are $D_{e,kk}^{s,m}=\sum_{i=1}^{N_{m}}H_{d,i,k}^{s,m}$. By summing the dynamic membership strengths of all nodes within a hyperedge, the effective response weight of that hyperedge is dynamically quantified, and degree normalization suppresses the interference that hyperedges of different cardinalities would otherwise introduce to feature magnitudes. To distinguish the discriminative contribution of each spatial hyperedge, a hyperedge attention mechanism is introduced: a learnable projection matrix $W_{att}^{s,m}\in {\mathbb{R}}^{d\times 1}$ scores each hyperedge, and Softmax normalizes the scores:
  (24) $$\alpha^{s,m}=\mathrm{Softmax}\;\left(E^{s,m}W_{att}^{s,m}\right)\in {\mathbb{R}}^{N\times K_{m}\times 1},\;\;\;\;{\tilde{E}}^{s,m}=\alpha^{s,m}⊙E^{s,m}\in {\mathbb{R}}^{N\times K_{m}\times d}$$
- **Hyperedge-to-node redistribution.** The weighted hyperedge features are broadcast back to nodes via $H_{d}^{s,m}$, and node features are updated through a learnable projection matrix $W_{n}^{s,m}\in {\mathbb{R}}^{d\times d}$, a BN layer, and a Gaussian Error Linear Unit (GELU) activation, with a residual connection and layer normalization to preserve the input:
  (25) $${\hat{Z}}^{m}=\mathrm{LayerNorm}\;\left(Z^{m}+\mathrm{GELU}\;\left(\mathrm{BN}\;\left(H_{d}^{s,m}{\tilde{E}}^{s,m}W_{n}^{s,m}\right)\right)\right)\in {\mathbb{R}}^{N\times N_{m}\times d}$$
  Each node feature in ${\hat{Z}}^{m}$ now encodes the high-order whole-brain spatial synergy within its frequency band, providing a spatially contextualized representation for the subsequent frequency hypergraph convolution.

(c). Frequency Hypergraph Convolution

Frequency hypergraph convolution takes the spatial hypergraph output ${\hat{Z}}^{m}\in {\mathbb{R}}^{N\times N_{m}\times d}$ as input and, building on the fused spatial context, further models high-order cross-band coupling within each channel. It proceeds through the same two alternating stages.

- **Node-to-hyperedge aggregation.** Using the dynamic frequency association matrix $H_{d}^{f,m}$ and the dynamic hyperedge degree matrix $D_{e}^{f,m}\in {\mathbb{R}}^{N\times C_{m}\times C_{m}}$, degree-normalized hypergraph convolution is performed:
  (26) $$E^{f,m}={\left(D_{e}^{f,m}\right)}^{-1}{\left(H_{d}^{f,m}\right)}^{⊤}{\hat{Z}}^{m}\in {\mathbb{R}}^{N\times C_{m}\times d}$$
  where $D_{e,cc}^{f,m}=\sum_{i=1}^{N_{m}}H_{d,i,c}^{f,m}$. A frequency hyperedge attention mechanism is introduced to distinguish the discriminative contributions of different frequency hyperedges: a learnable projection matrix $W_{att}^{f,m}\in {\mathbb{R}}^{d\times 1}$ scores each hyperedge, and Softmax normalizes the scores:
  (27) $$\alpha^{f,m}=\mathrm{Softmax}\;\left(E^{f,m}W_{att}^{f,m}\right)\in {\mathbb{R}}^{N\times C_{m}\times 1},\;\;\;\;{\tilde{E}}^{f,m}=\alpha^{f,m}⊙E^{f,m}\in {\mathbb{R}}^{N\times C_{m}\times d}$$
- **Hyperedge-to-node redistribution.** The weighted hyperedge features are broadcast back to nodes via $H_{d}^{f,m}$, and node features are updated through a learnable projection matrix $W_{n}^{f,m}\in {\mathbb{R}}^{d\times d}$, a BN layer, and a GELU activation, with a residual connection and layer normalization:
  (28) $${\tilde{Z}}^{m}=\mathrm{LayerNorm}\;\left({\hat{Z}}^{m}+\mathrm{GELU}\;\left(\mathrm{BN}\;\left(H_{d}^{f,m}{\tilde{E}}^{f,m}W_{n}^{f,m}\right)\right)\right)\in {\mathbb{R}}^{N\times N_{m}\times d}$$
  Each node feature in ${\tilde{Z}}^{m}$ simultaneously encodes cross-channel spatial synergy within its frequency band and cross-band coupling within its single channel, achieving hierarchical high-order representation fusion across both the spatial and frequency dimensions.

(d). Skip Connections and Multi-view Pooling

The serial unit of one spatial hypergraph convolution followed by one frequency hypergraph convolution is defined as an SF-Block, stacked for L layers with independent parameters. To alleviate feature over-smoothing in deep hypergraph networks and preserve multi-scale semantics accumulated at different propagation depths, we introduce skip connections that concatenate the output features $\left\{{\tilde{Z}}^{m,(1)},{\tilde{Z}}^{m,(2)},\ldots ,{\tilde{Z}}^{m,(L)}\right\}$ of all SF-Blocks along the feature dimension and then project them back to the original feature dimension d via a linear mapping:

(29) $$Z_{SC}^{m}=W_{SC}^{m}\cdot F\;\left(\left[{\tilde{Z}}^{m,(1)};\;{\tilde{Z}}^{m,(2)};\;\ldots ;\;{\tilde{Z}}^{m,(L)}\right]\right)\in {\mathbb{R}}^{N\times N_{m}\times d}$$

where $W_{SC}^{m}\in {\mathbb{R}}^{d\times (L\cdot d)}$ is a learnable linear projection matrix and F(⋅) is a flattening operator. The skip connections allow the model to retain both shallow local frequency-channel activation patterns and deep high-order cross-frequency topological synergy, effectively countering the node feature homogenization that tends to accompany deeper architectures.

After skip-connection fusion, the node features $Z_{SC}^{m}\in {\mathbb{R}}^{N\times N_{m}\times d}$ are reshaped to ${\mathbb{R}}^{N\times K_{m}\times C_{m}\times d}$, and global discriminative representations are extracted in parallel along the frequency and spatial dimensions.

- Frequency pooling averages over the channel dimension, flattens the result, and applies a linear projection to obtain a frequency-aggregated representation that captures band-level global discriminative semantics across channels for a given neural oscillatory rhythm:
  (30) $$r_{b}^{m}=W_{b}^{m}\cdot F\;\left(\frac{1}{C_{m}}\sum_{c=1}^{C_{m}}Z_{SC}^{m,(k,c)}\right)\in {\mathbb{R}}^{N\times d}$$
- Channel pooling averages over the frequency dimension, flattens the result, and applies a linear projection to obtain a channel-aggregated representation that captures spatial-level global discriminative semantics across frequency bands for a given brain region:
  (31) $$r_{c}^{m}=W_{c}^{m}\cdot F\;\left(\frac{1}{K_{m}}\sum_{k=1}^{K_{m}}Z_{SC}^{m,(k,c)}\right)\in {\mathbb{R}}^{N\times d}$$

The two views are concatenated along the feature dimension and fused via a linear projection to yield the modality-specific one-dimensional high-order discriminative feature $R^{m}\in {\mathbb{R}}^{N\times d}$:

(32) $$R^{m}=W_{r}^{m}\cdot \left[r_{b}^{m};\;r_{c}^{m}\right]\in {\mathbb{R}}^{N\times d}$$

where $W_{r}^{m}\in {\mathbb{R}}^{d\times 2d}$ is a learnable fusion projection matrix. Frequency pooling and channel pooling supply complementary discriminative semantics at the band level and the channel level, respectively, jointly ensuring that the final emotional feature retains discriminative power in both the spatial and frequency dimensions. After independent SF-AHGC processing, the EEG and fNIRS branches output high-order discriminative features $R^{e}\in {\mathbb{R}}^{N\times d}$ and $R^{f}\in {\mathbb{R}}^{N\times d}$, respectively.

#### 2.5.4. Cross-Modal Attention Fusion

CMAF exploits the complementarity of EEG and fNIRS by casting the fNIRS representation $R^{f}\in {\mathbb{R}}^{N\times d}$ as the query and the EEG representation $R^{e}\in {\mathbb{R}}^{N\times d}$ as both key and value. These are mapped to a shared latent space through independent learnable linear projections:

(33) $$Q=R^{f}W_{q}\in {\mathbb{R}}^{N\times d},\;\;\;\;K=R^{e}W_{k}\in {\mathbb{R}}^{N\times d},\;\;\;\;V=R^{e}W_{v}\in {\mathbb{R}}^{N\times d}$$

where $W_{q},W_{k},W_{v}\in {\mathbb{R}}^{d\times d}$ are learnable projection matrices. Using fNIRS as the query exploits its spatial indication of emotion-related brain region activation to guide the attention mechanism in selectively extracting, from the EEG representation, neural oscillatory components semantically linked to the current hemodynamic activation—an explicit neurophysiological grounding for the cross-modal fusion. The cross-modal response is computed via scaled dot-product attention:

(34) $$R_{a}=\mathrm{Softmax}\;\left(\frac{QK^{⊤}}{\sqrt{d}}\right)V\in {\mathbb{R}}^{N\times d}$$

The attention output $R_{a}$ is concatenated with the original EEG representation $R^{e}$ along the feature dimension, and the final joint emotional representation is obtained through a learnable fusion projection matrix $W_{f}\in {\mathbb{R}}^{d\times 2d}$ and a GELU activation:

(35) $$R_{f}=\mathrm{GELU}\;\left(W_{f}\left[R^{e};R_{a}\right]\right)\in {\mathbb{R}}^{N\times d}$$

The joint representation $R_{f}$ is then passed to an emotion classifier for category prediction. The classifier stacks a linear projection layer, a BN layer, a Rectified Linear Unit (ReLU) activation, a Dropout regularization layer, and an output linear layer:

(36) $$\hat{y}=\mathrm{Softmax}\;\left(W_{o}D\;\left(\mathrm{ReLU}\;\left(\mathrm{BN}\;\left(W_{c}R_{f}\right)\right)\right)\right)\in {\mathbb{R}}^{N\times C}$$

where $W_{c}\in {\mathbb{R}}^{d_{h}\times d}$ is the hidden-layer projection weight matrix with hidden dimension $d_{h}$; $W_{o}\in {\mathbb{R}}^{C\times d_{h}}$ is the output-layer weight matrix; $\hat{y}$ is the model-predicted probability distribution over emotion categories; and D(⋅) denotes Dropout.

The training procedure of SF-HGNN is summarized in Algorithm 1.
**Algorithm 1.** SF-HGNN Forward Propagation
**Input:** EEG signal $x^{e}$, fNIRS signal $x^{f}$
**Output:** Predicted emotion probability vector $\hat{y}$1**for** each modality m∈{e,f} **do**2
Compute learnable frequency boundaries $\left\{b_{k}^{m}\right\}$ and soft masks $\left\{m_{k}^{m}\right\}$;3
$X_{k}^{m}=\mathrm{IRFFT}(\mathrm{RFFT}(x^{m})\cdot m_{k}^{m})$ for k=1 to $K_{m}$;4
Concatenate into multi-band tensor $X^{m}$;5
$Z^{m}=\mathrm{MTC}(X^{m})$;6
Initialize structural masks $M^{s,m}$ and $M^{f,m}$;7
**for** l=1 **to** L **do**8

Compute dynamic spatial association matrix $H_{d}^{s,m}$;9

$Z^{m}\leftarrow \mathrm{LayerNorm}(Z^{m}+\mathrm{SHGConv}(Z^{m};H_{d}^{s,m}))$;10

Compute dynamic frequency association matrix $H_{d}^{f,m}$;11

$Z^{\left(l\right)}\leftarrow \mathrm{LayerNorm}(Z^{m}+\mathrm{FHGConv}(Z^{m};H_{d}^{f,m}))$;12

$Z^{m}\leftarrow Z^{\left(l\right)}$;13
$Z_{SC}^{m}\leftarrow \mathrm{SkipFuse}(Z^{\left(1\right)},\ldots ,Z^{\left(L\right)})$;14
$R^{m}\leftarrow \mathrm{MultiViewPool}(Z_{SC}^{m})$;15$R_{f}\leftarrow \mathrm{CMAF}(R^{e},R^{f})$;16$\hat{y}\leftarrow \mathrm{Softmax}(\mathrm{MLP}(R_{f}))$;17**return** $\hat{y}$

## 3. Results Analysis and Comparison

To comprehensively evaluate the performance of the proposed model, we conducted subject-dependent experiments, subject-independent experiments, and ablation experiments, together with t-distributed Stochastic Neighbor Embedding (t-SNE) analysis of the adaptive association matrices and feature distributions. The subject-dependent experiments employed five-fold cross-validation at the trial level to partition training and test sets, while the subject-independent experiments adopted Leave-One-Subject-Out (LOSO) cross-validation for cross-subject assessment [36]. Both experiments used Accuracy (ACC) with its standard deviation (STD) and macro F1-score (F1) as quantitative metrics of classification performance, computed as follows:

(37) $$\left\{\begin{array}{l} ACC=\frac{1}{N}\sum_{i=1}^{C}TP_{i},\\ F1_{macro}=\frac{1}{C}\sum_{i=1}^{C}\frac{2TP_{i}}{2TP_{i}+FP_{i}+FN_{i}}. \end{array}\right.$$

where $TP_{i}$ denotes the number of true positives for class i, i.e., samples correctly predicted as belonging to that class; $FP_{i}$ and $FN_{i}$ are the numbers of false positives and false negatives for class i, respectively. N is the total number of samples, and C is the number of emotion categories.

Table 1 summarizes the hyperparameters of SF-HGNN, covering the numbers and initial boundaries of the EEG and fNIRS frequency bands, the temperature parameters of the transition bands, the kernel sizes of the Multi-scale Temporal Convolution, the hidden dimension, and the number of hypergraph convolution layers; it also lists the training hyperparameters, including the optimizer, learning rate, weight decay, label smoothing coefficient, and random seed.

### 3.1. Subject-Dependent Experiment

The subject-dependent (SD) experiment employed five-fold cross-validation to partition training and test sets. To prevent data leakage, the dataset was divided with trial as the atomic unit, ensuring that all time segments belonging to a given trial resided exclusively in either the training set or the test set.

Table 2 summarizes the average SD performance of SF-HGNN and all baseline methods on the ENTER dataset. SF-HGNN achieves the best results across all metrics, with ACC, STD, and F1 reaching 82.94%, 3.28%, and 82.97%, respectively. Compared with traditional machine learning methods, SF-HGNN outperforms SVM by 31.81% and TCN by 21.54% in ACC, underscoring the advantage of end-to-end deep feature extraction over handcrafted features. Relative to baselines specifically designed for EEG-fNIRS multimodal tasks, SF-HGNN surpasses EFMLNet by 8.12%, FGANet by 9.70%, and E-FNet by 6.55% in ACC. Conformer also delivers competitive emotion recognition results with an ACC of 80.66%, yet still trails SF-HGNN by 2.28% in ACC. Compared with the strongest baseline STA-Net, SF-HGNN improves ACC by 1.77% and F1 by 1.82%, confirming its superiority in subject-dependent emotion recognition. These results indicate that SF-HGNN effectively captures high-order interactive information between EEG and fNIRS across both frequency and spatial dimensions, thereby yielding more discriminative EEG-fNIRS emotional representations.

Furthermore, SF-HGNN contains only 1.29 M trainable parameters. It outperforms both Conformer and STA-Net at a lower parameter count, suggesting that its performance gains derive from effective modeling of higher-order EEG-fNIRS interactions rather than from model scaling. Although SF-HGNN is larger than lightweight alternatives such as EFMLNet, FGANet, and E-FNet, its substantial performance margin demonstrates that adequate model capacity enhances the learning of complex brain network topology and multimodal correlations. Overall, SF-HGNN achieves efficient, highly discriminative EEG-fNIRS emotional representation learning at modest model complexity.

**Figure 7:**
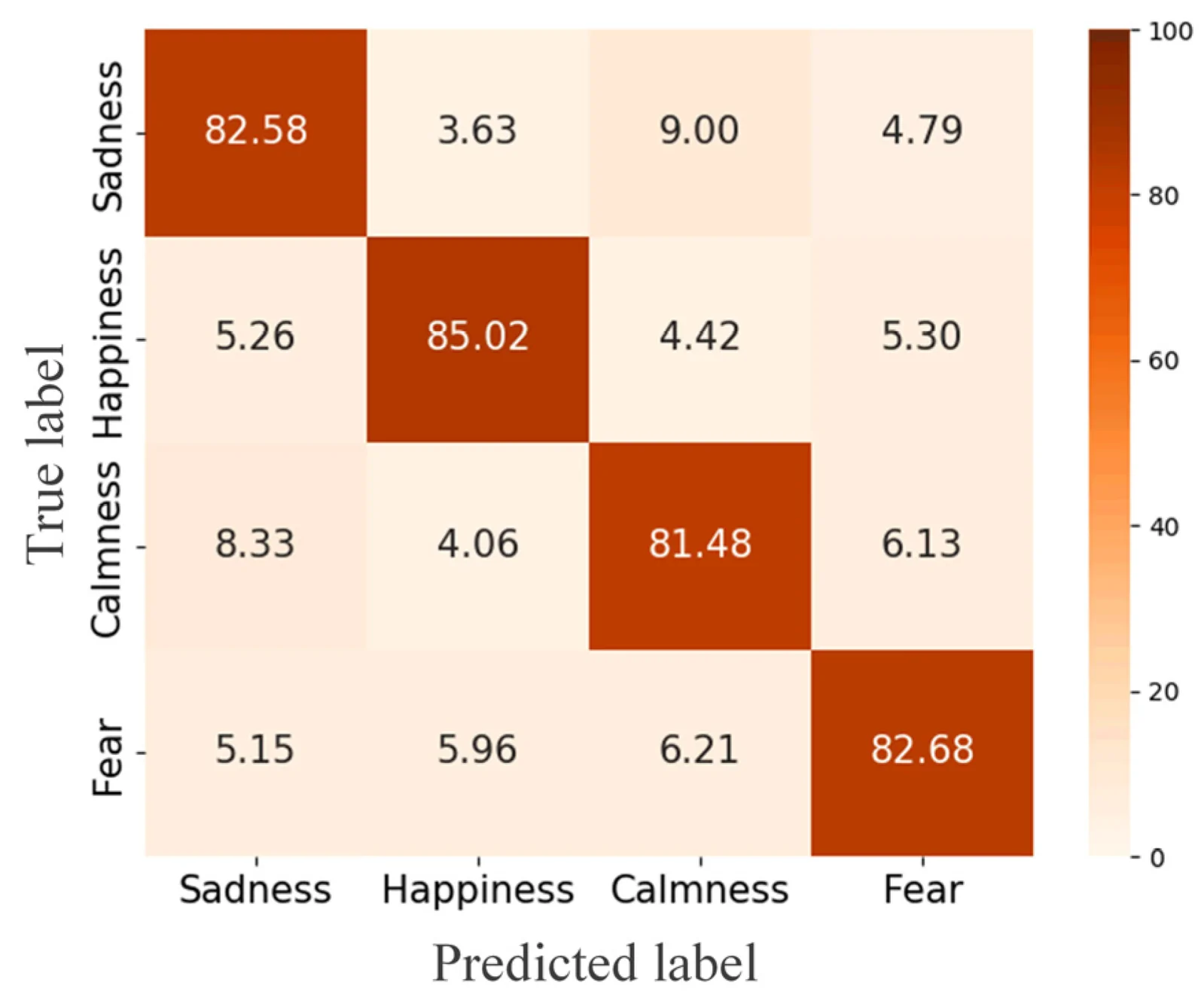
Confusion matrices of SF-HGNN in the SD emotion recognition experiment.

Figure 7 presents the confusion matrices of SF-HGNN in the SD experiment on the ENTER dataset. SF-HGNN achieves the highest recognition accuracy for sadness, while calm shows the lowest performance. This is mainly because calm lies at the center of the valence-arousal space, leading to overlapping feature distributions with other emotions.

### 3.2. Subject-Independent Experiment

To evaluate the performance of SF-HGNN under cross-subject generalization, we further conducted subject-independent (SI) experiments using the LOSO cross-validation strategy. In each fold, the complete EEG and fNIRS data of one subject were held out as the test set, and the data of the remaining subjects were used for model training.

Table 3 summarizes the average SI performance of SF-HGNN and baseline methods on the ENTER dataset. SF-HGNN achieves the best overall performance, with ACC, STD, and F1 reaching 68.85%, 8.53%, and 68.79%, respectively. In comparison, SVM obtains only 42.75% ACC, demonstrating limited cross-subject emotion recognition capability. TCN and EEGNet achieve ACC values of 47.89% and 48.91%, respectively, indicating that traditional machine learning methods and classical deep models still suffer from insufficient generalization under substantial inter-individual variability. Among EEG-fNIRS multimodal approaches, FGANet achieves 60.83% ACC but remains 8.02% lower than SF-HGNN, suggesting that attention-based methods without explicit high-order brain network modeling are insufficient for cross-subject scenarios. STA-Net, as a strong baseline with temporal modeling capability, achieves 67.26% ACC. Compared with STA-Net, SF-HGNN further improves ACC and F1 by 1.59% and 1.54%, respectively, demonstrating its effectiveness in capturing cross-modal high-order brain network structures and improving inter-subject generalization.

**Figure 8:**
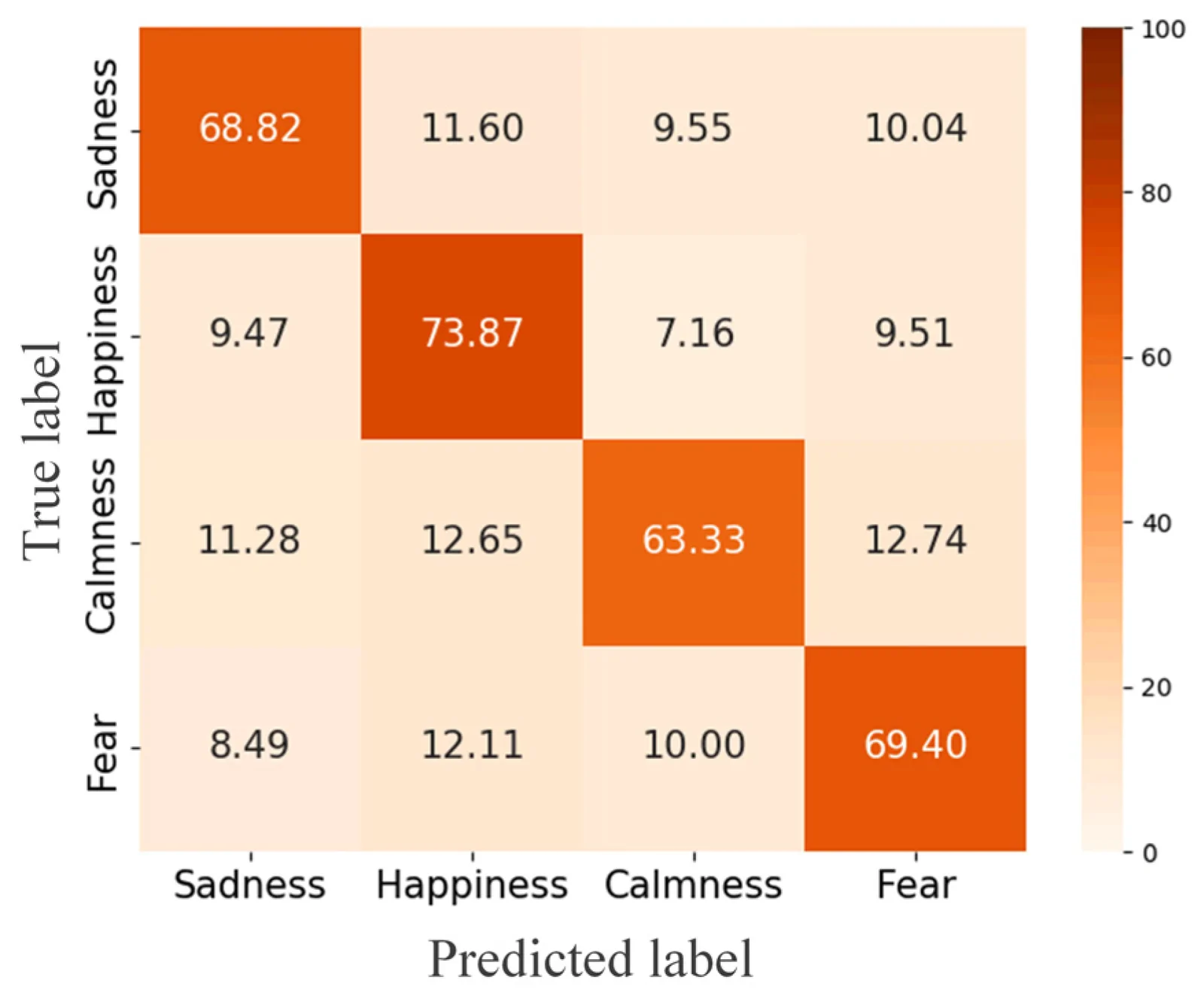
Confusion matrices of SF-HGNN in the SI emotion recognition experiment.

Figure 8 presents the confusion matrices of SF-HGNN in the SI experiment on the ENTER dataset. Consistent with the SD results, the calm category again exhibits lower recognition accuracy than other emotion categories, further corroborating the inherent difficulty of recognizing this category under cross-subject settings.

### 3.3. EEG and fNIRS Frequency Band Visualization

**Figure 9:**
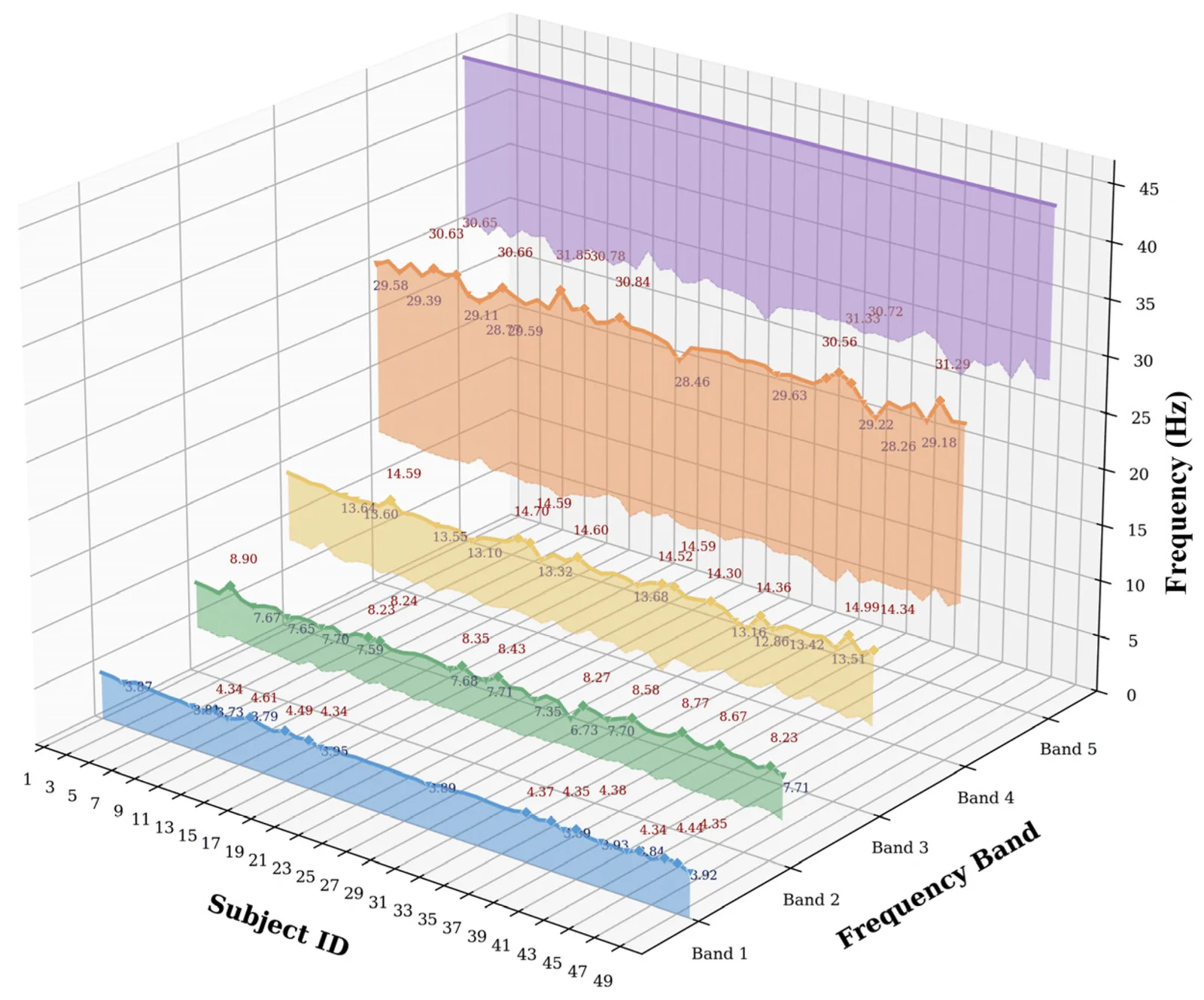
EEG frequency band intervals of different subjects.

To answer RQ1, we performed a statistical analysis of the EEG and fNIRS signals of all 50 subjects in the emotion recognition experiments to investigate the frequency band ranges learned by DyFreBD. The initial global frequency bands for EEG signals were set as 0–4 Hz, 4–8 Hz, 8–14 Hz, 14–30 Hz, and 30–45 Hz. As shown in Figure 9, after data-driven adaptive learning, the frequency band boundaries exhibit pronounced inter-individual variability. In Band 1, Subject 14 has the largest upper bound at 4.61 Hz, whereas Subject 10 has the smallest at 3.73 Hz. In Band 2, Subject 4 has the largest upper bound at 8.90 Hz, and Subject 33 the smallest at 6.73 Hz. In Band 3, the upper bounds are generally elevated, with the majority exceeding 13 Hz; only Subject 40 has an upper bound slightly below this value at 12.86 Hz. In Band 4, Subject 17 has the highest upper bound at 31.85 Hz, while Subject 43 has the lowest at 28.26 Hz. These results demonstrate that the adaptive frequency band boundary learning method can dynamically adjust to individual-specific neural oscillatory characteristics, thereby capturing individual brain activity with greater precision.

**Figure 10:**
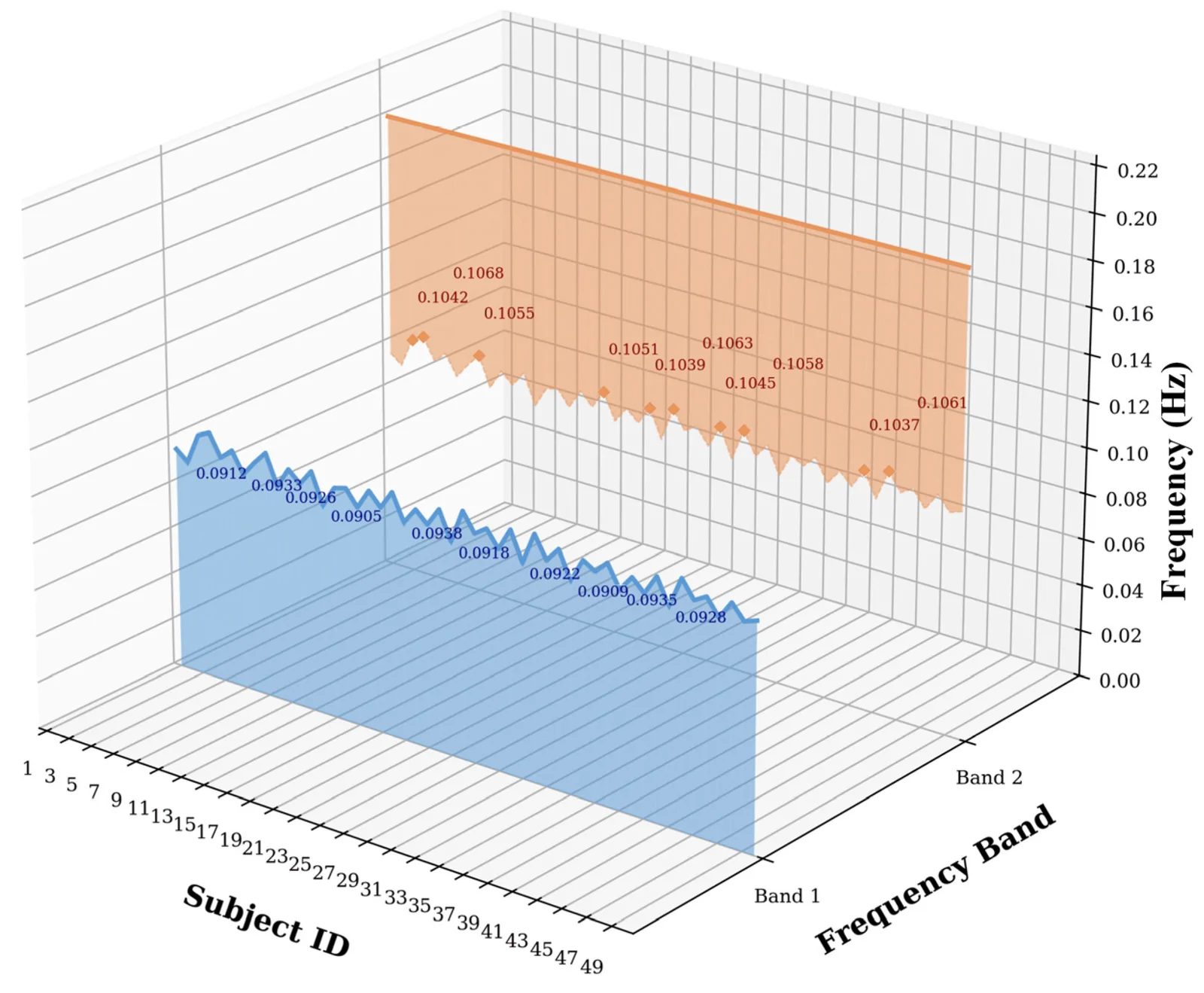
fNIRS frequency band intervals of different subjects.

The initial global frequency bands for fNIRS signals were set as 0.01–0.10 Hz and 0.10–0.20 Hz. As shown in Figure 10, after data-driven adaptive learning, the frequency band division boundaries of different subjects exhibit a high degree of consistency with only subtle inter-individual variation. In Band 1, Subject 4 has the largest upper bound at 0.1068 Hz, while Subject 14 has the smallest at 0.0905 Hz. The frequency band cut-points for the vast majority of subjects are tightly clustered within the 0.095–0.105 Hz range. These results indicate that the adaptive frequency band boundary learning method can effectively filter out subtle individual physiological noise and adaptively and precisely isolate the frequency band ranges that reflect cerebral hemodynamic characteristics.

### 3.4. Ablation Study

To answer RQ2, we performed component-wise ablation experiments under both the SD and SI evaluation protocols to verify the contribution of each component of SF-HGNN. Table 4 reports the results of each ablation variant, where reversed CMAF denotes the complete SF-HGNN model with EEG as the query and fNIRS as the key and value; w/o DyFreBD denotes the removal of the Dynamic Frequency Band Decomposition module; w/o MTC denotes the removal of the Multi-scale Temporal Convolution module; w/o SF-AHGC denotes the removal of the Spatial-Frequency Adaptive Hypergraph Convolution module; and w/o CMAF denotes the removal of the Cross-Modal Attention Fusion module.

In the SD experiment, the complete SF-HGNN achieves the optimal ACC of 82.94%. When the query and key-value roles in CMAF are reversed, the ACC of SF-HGNN decreases by 1.03%. The removal of SF-AHGC causes the largest ACC drop, from 82.94% to 80.68%, a decrease of 2.26%, indicating that the Spatial-Frequency Adaptive Hypergraph Convolution module is the most critical component for capturing high-order collective interactions of EEG and fNIRS across frequency and spatial dimensions. Removing CMAF incurs an ACC decrease of 1.39%, demonstrating that the cross-modal attention mechanism with fNIRS as query and EEG as key-value pair contributes substantially to fusing the complementary information of the two modalities. Removing MTC leads to an ACC decrease of 1.07%, confirming that Multi-scale Temporal Convolution makes a material contribution to emotion feature extraction through its joint modeling of local transient activation patterns and long-range rhythmic patterns. By contrast, removing DyFreBD causes only a 0.18% ACC decrease, the smallest drop among all ablations. However, given that DyFreBD is fundamentally a preprocessing-level frequency-adaptive decomposition module, its contribution primarily lies in supplying higher-quality frequency band partitions to downstream modules.

In the SI experiment, the overall ablation trend is consistent with that of the SD experiment. When the query and key-value roles in CMAF are reversed, the ACC of SF-HGNN decreases by 0.66%. The removal of SF-AHGC causes the largest ACC drop, from 68.85% to 66.94%, a decrease of 1.91%, further confirming that high-order hypergraph modeling is equally critical for cross-subject generalization. Removing CMAF leads to a 0.97% decrease in ACC, removing MTC leads to a 0.75% decrease, and removing DyFreBD leads to a 0.12% decrease.

### 3.5. Association Matrix Visualization

**Figure 11:**
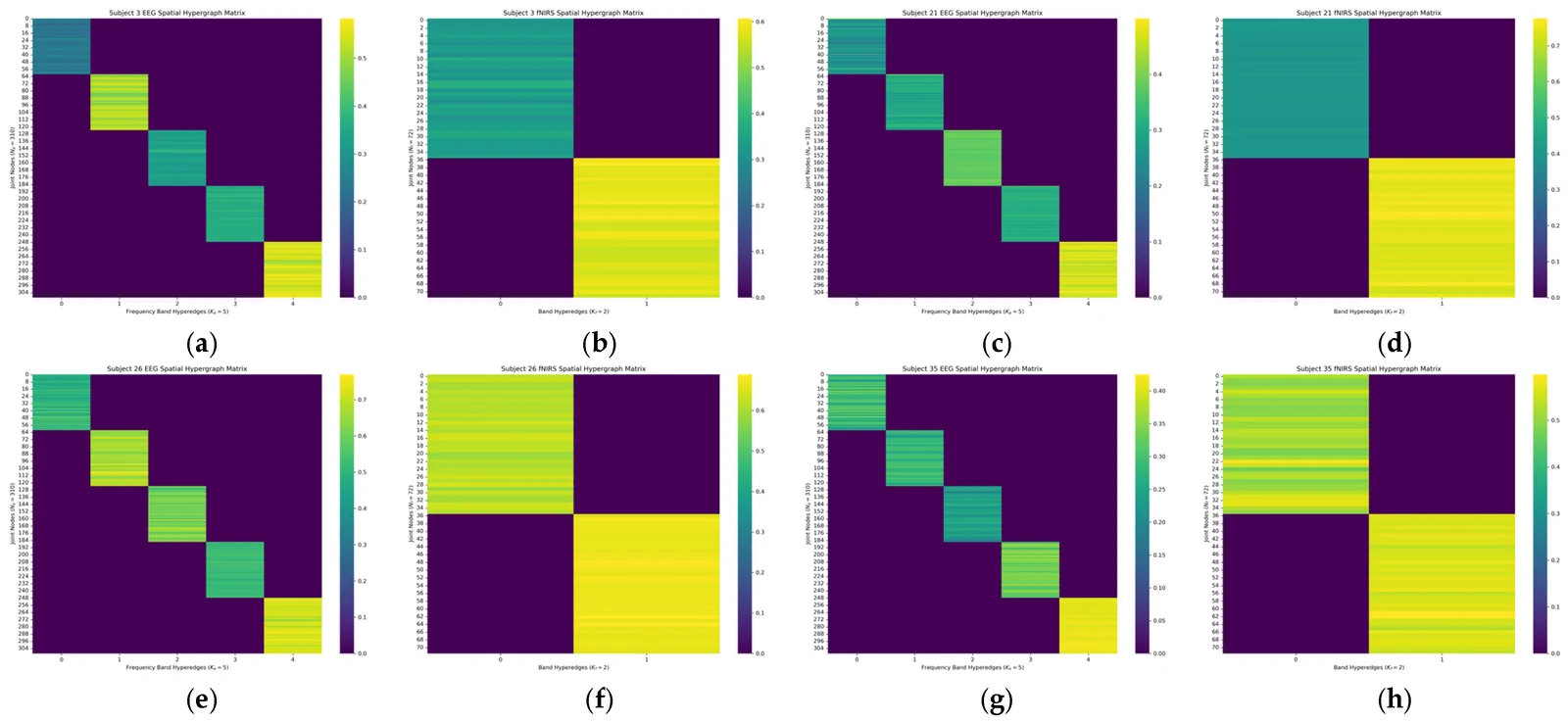
Spatial hypergraph association matrix analysis. (**a**,**b**), (**c**,**d**), (**e**,**f**), and (**g**,**h**) correspond to the EEG and fNIRS spatial association matrices of Subjects 3, 21, 26, and 35, respectively.

**Figure 12:**
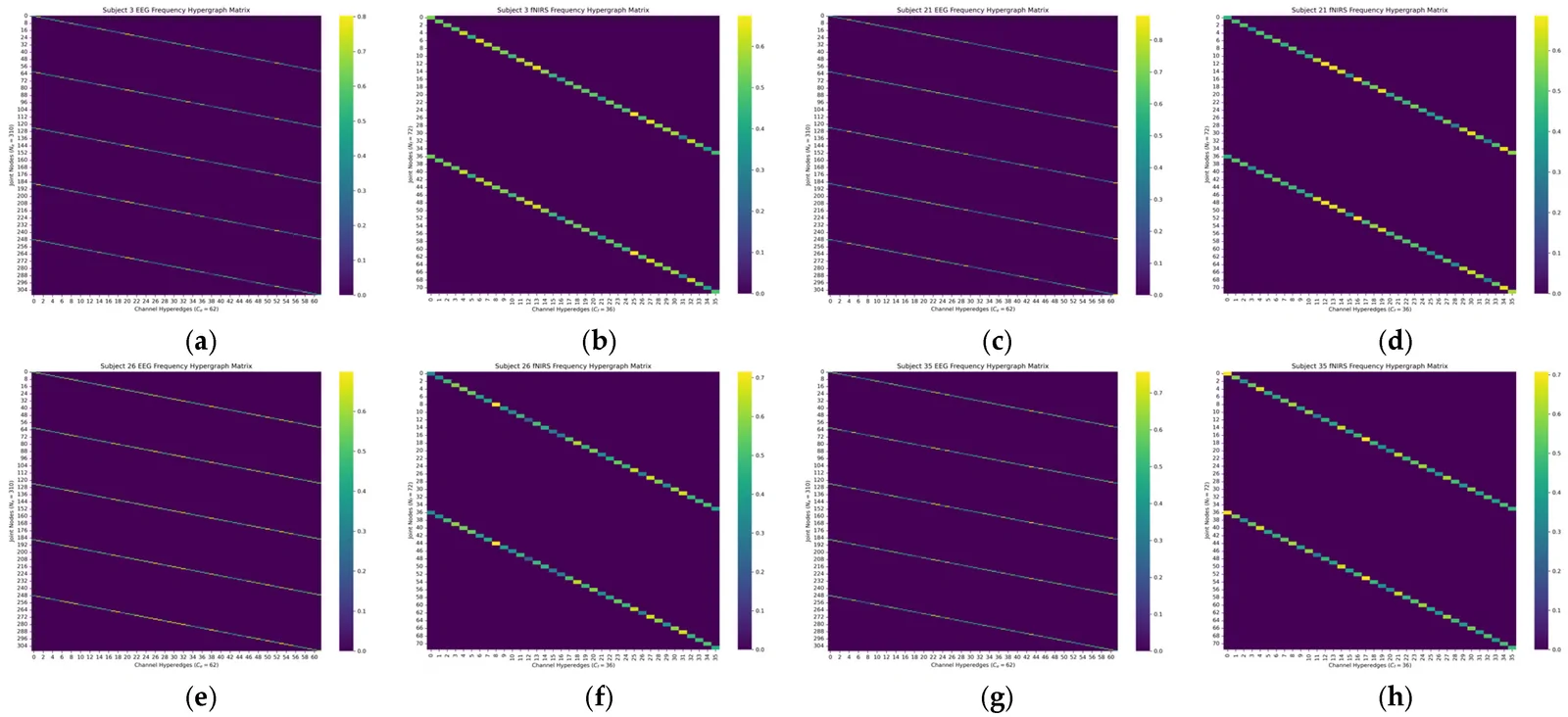
Frequency hypergraph association matrix analysis. (**a**,**b**), (**c**,**d**), (**e**,**f**), and (**g**,**h**) correspond to the EEG and fNIRS frequency association matrices of Subjects 3, 21, 26, and 35, respectively.

To answer RQ3, we randomly selected four subjects (3, 21, 26, and 35) and performed interpretability analysis on their spatial and frequency hypergraph association matrices to further investigate the high-order spatial-frequency interactions learned by the SF-AHGC module. To mitigate the impact of single-sample fluctuations, the visualization results were smoothed by sample averaging. In the matrices, rows correspond to joint nodes of the hypergraph, columns correspond to frequency-band hyperedges or channel hyperedges, and color intensity indicates the weight of a node with respect to a given hyperedge. For the EEG branch, the dimensions of the spatial and frequency hypergraph association matrices are $N_{e}\times K_{e}$ and $N_{e}\times C_{e}$, respectively; for the fNIRS branch, the corresponding dimensions are $N_{f}\times K_{f}$ and $N_{f}\times C_{f}$. Figure 11 and Figure 12 present the spatial and frequency hypergraph association matrices of these four subjects, respectively.

As shown in Figure 11a,c,e,g, the EEG spatial hypergraph association matrices display pronounced frequency band selectivity, with markedly different dynamic membership weights across frequency bands. Bands 1, 3, and 4 have relatively low overall weights, whereas Band 5 exhibits higher dynamic membership weights, suggesting that this band carries greater discriminative contribution during emotion recognition. This phenomenon indicates that different frequency components correspond to distinct neural oscillatory mechanisms and that their capacity to represent emotional states differs appreciably. As shown in Figure 11b,d,f,h, the fNIRS spatial hypergraph association matrices likewise exhibit non-uniform distributions across frequency bands: Band 1 has generally lower dynamic membership weights, while Band 2 exhibits stronger response intensity, indicating that different hemodynamic frequency bands also contribute differentially to emotion recognition. When considered jointly across the two modalities, the high-frequency bands tend to carry higher dynamic membership weights, suggesting that they encode richer emotion-discriminative information. Furthermore, in both the EEG and fNIRS spatial hypergraph association matrices, the dynamic membership weights of different channels within the same frequency band also vary markedly, indicating that the contributions of different brain regions to emotion processing within the same frequency range are not uniform. These findings further validate that SF-AHGC adaptively learns high-order cross-channel spatial synergistic relationships within each frequency band and dynamically adjusts the association strength between nodes and spatial hyperedges according to their importance, thereby effectively enhancing the model’s capacity to capture spatial discriminative features.

As shown in Figure 12a,c,e,g, the EEG frequency hypergraph association matrices form five continuously distributed high-response diagonal bands, corresponding to the cross-frequency coupling relationships established between the $K_{e}$ frequency band node clusters and all $C_{e}$ channel hyperedges. The dynamic membership weights differ markedly across channels. Specifically, Channels 20 and 52 for Subject 3, Channels 4 and 61 for Subject 21, Channels 26 and 32 for Subject 26, and Channels 22 and 43 for Subject 35 all exhibit relatively high dynamic membership weights, indicating that these brain regions play a more prominent role in cross-frequency information modeling. As shown in Figure 12b,d,f,h, the fNIRS frequency hypergraph association matrices form two high-response bands distributed along the diagonal, corresponding to the continuous cross-frequency coupling relationships between the $K_{f}$ frequency band node clusters and all $C_{f}$ channel hyperedges. Once again, the dynamic membership weights vary substantially across channels. Channels 12, 13, and 24 for Subject 3; Channels 12, 13, and 16 for Subject 21; Channels 8, 27, and 31 for Subject 26; and Channels 4, 13, and 17 for Subject 35 all carry relatively high dynamic membership weights, suggesting that these regions possess greater emotion-discriminative contribution in hemodynamic cross-frequency coupling. Moreover, in both the EEG and fNIRS frequency hypergraph association matrices, the dynamic membership weights of different frequency-band nodes within the same channel also differ markedly, indicating that the extent to which a given brain region participates in emotion processing varies across frequency ranges. These results demonstrate that SF-AHGC adaptively learns high-order cross-band coupling relationships within each brain region and dynamically adjusts the association strength between nodes and frequency hyperedges according to the importance of different frequency components for emotion recognition, thereby fully mining cross-frequency synergistic information and enhancing the model’s frequency discriminative capability.

### 3.6. t-SNE Visualization

**Figure 13:**
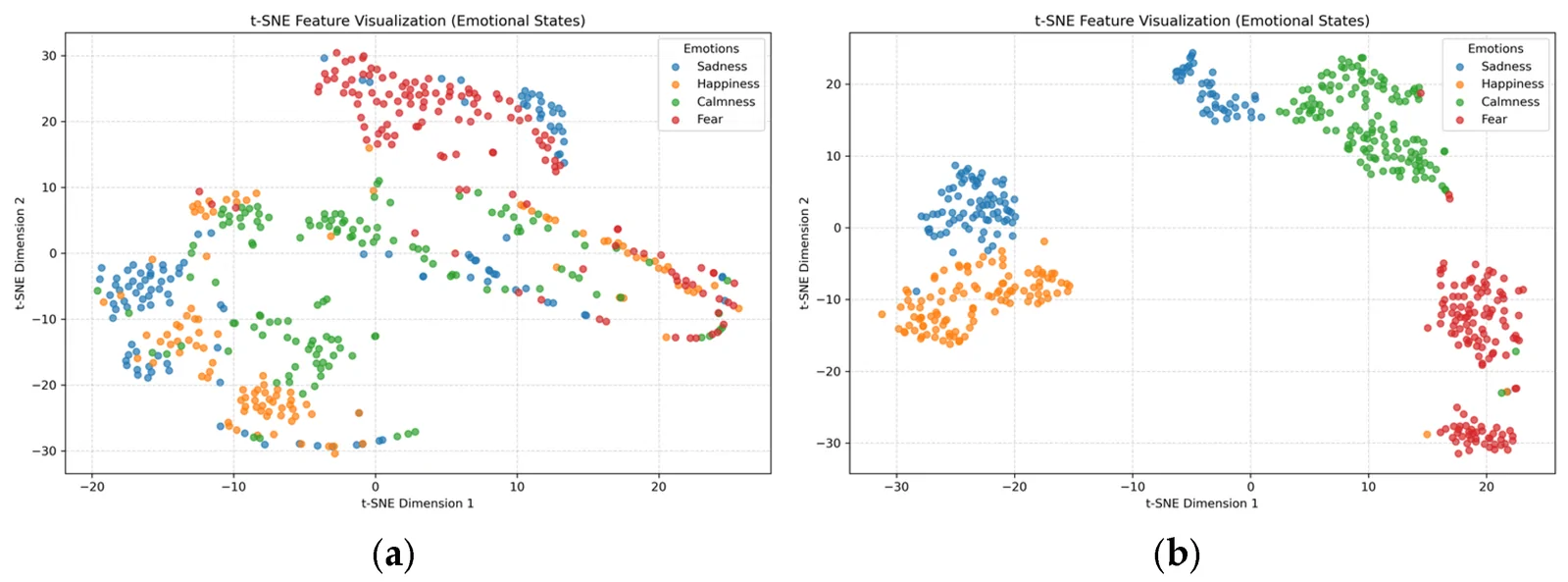
t-SNE analysis of initial and final EEG-fNIRS embeddings. (**a**) Initial embeddings. (**b**) Final embeddings.

To intuitively assess the emotion-discriminative capability of SF-AHGC and its feature representation learning effectiveness, we performed t-SNE visualization analysis on the feature distributions before and after model training. Figure 13a,b display the original EEG-fNIRS feature embeddings and the feature embeddings optimized by SF-HGNN, respectively. Prior to training, the initial EEG-fNIRS features are relatively scattered in the low-dimensional space: samples of the same emotion category fail to form tight clusters, and a degree of overlap exists among different categories. After SF-HGNN training, the learned EEG-fNIRS features exhibit more compact intra-class aggregation and more pronounced inter-class separation, demonstrating that SF-HGNN effectively mines the high-order interactive information and discriminative features between EEG and fNIRS signals and improves the separability of emotion categories. Notably, the sadness category exhibits two relatively independent sub-clusters in the feature space. This may be attributed to the subjective nature and individual variability of sadness, which give rise to distinct neurophysiological representations in subjects’ EEG oscillations and fNIRS hemodynamic responses. Nevertheless, these two sub-clusters maintain clear decision boundaries with other emotion categories without cross-category confusion. These results indicate that SF-HGNN effectively captures the higher-order interactive information and discriminative features between EEG and fNIRS signals, accurately preserving fine-grained physiological expression characteristics while achieving decoupling of high-dimensional emotional representations.

## 4. Discussion

### 4.1. Main Findings

This study proposes an EEG-fNIRS emotion recognition framework based on a spatial-frequency Hypergraph Neural Network and systematically validates it on the ENTER dataset. The experimental results show that SF-HGNN outperforms existing methods under both subject-dependent and subject-independent evaluation protocols. The ablation experiments confirm the effectiveness of each module, with SF-AHGC making the most critical contribution, while the contribution of DyFreBD is relatively small. The visualization analyses further show that the learned frequency band boundaries exhibit obvious individual differences and that the hypergraph association matrices present structured patterns corresponding to frequency bands and channels, indicating that SF-HGNN not only achieves superior recognition performance but also possesses neurophysiological interpretability.

### 4.2. Comparison with Existing Methods

Compared with traditional methods that rely on fixed frequency band partitioning, DyFreBD treats frequency band boundaries as learnable parameters, avoiding the fragmentation or aliasing of frequency components at fixed boundaries and enabling the spectral decomposition to adapt to inter-individual differences in neural oscillations. Although the ablation experiments show that the performance gain brought by DyFreBD is relatively limited, its role in cross-subject scenarios should not be overlooked, which is consistent with previous findings on individual differences in frequency characteristics [11,14]. Compared with graph-based methods built on pairwise connections, such as ST-DGAT, SF-AHGC models multi-channel synergistic activation and cross-frequency coupling as high-order holistic relationships through spatial and frequency hypergraphs. In the ablation experiments, removing SF-AHGC causes the largest performance drop, indicating that high-order brain network relationship modeling is the core source of performance improvement. This is consistent with the conclusion that hypergraphs can more naturally characterize group-level synergistic activities in brain signal analysis [15,30]. Compared with methods specifically designed for EEG-fNIRS fusion, FGANet also adopts an attention mechanism that uses fNIRS to guide EEG, but it is still 8.02% lower than SF-HGNN in the cross-subject scenario, indicating that attention guidance alone without high-order structural modeling has limitations. STA-Net achieves strong baseline results with its temporal alignment capability; the advantage of SF-HGNN mainly lies in spatial-frequency high-order interaction modeling, and the two mechanisms are complementary and may be combined in future work. In addition, SF-HGNN achieves better performance than the larger Conformer and STA-Net with only 1.29 M parameters, indicating that its performance gain mainly stems from the architectural design rather than model scale.

### 4.3. Neurophysiological Interpretation

The frequency band visualization results show that the EEG frequency band boundaries learned by different subjects differ markedly (e.g., the upper bound of Band 1 ranges from 3.73 to 4.61 Hz), reflecting inter-individual differences in neural oscillatory rhythms, whereas the fNIRS frequency band boundaries are highly consistent, which is consistent with the physiological characteristics whereby hemodynamic responses are slower and show smaller individual differences. The association matrix analysis shows that EEG Band 5 carries higher dynamic membership weights in emotion recognition, suggesting that high-frequency neural oscillations may contain richer emotion-discriminative information, and fNIRS Band 2 also shows stronger response intensity. Meanwhile, some channels exhibit high cross-frequency coupling weights; the specific roles of these brain regions in emotion processing deserve further verification through source localization and other analyses. In the t-SNE visualization, the two sub-clusters of the sadness category may arise from the subjectivity and individual variability of sad emotions, but clear decision boundaries are still maintained among all emotion categories.

## 5. Conclusions

Motivated by the neurovascular coupling mechanism, we proposed an EEG-fNIRS emotion recognition framework based on SF-HGNN, designed to address the limitations of existing methods in fixed frequency band modeling and spatial-frequency high-order relationship learning. SF-HGNN constitutes an end-to-end learning framework that achieves in-depth exploitation of the complementary information carried by EEG and fNIRS through dynamic frequency decomposition, multi-scale temporal modeling, spatial-frequency hypergraph learning, and cross-modal fusion. Specifically, the DyFreBD module treats frequency band boundaries as learnable parameters and, via a differentiable filtering mechanism, adaptively discovers frequency ranges relevant to the emotion task, thereby mitigating the constraints imposed by fixed frequency band partitioning. The MTC module captures multi-scale dynamic features using distinct temporal receptive fields, modeling EEG neural oscillations and fNIRS hemodynamic changes across different time scales. The SF-AHGC module further constructs dynamic spatial and frequency hypergraphs to respectively model cross-channel spatial synergy within frequency bands and cross-band coupling within channels, thereby learning high-order interactive features from EEG-fNIRS signals. The CMAF module achieves effective complementary fusion between the two modalities through an attention mechanism, yielding more discriminative emotional representations. Experimental results on the ENTER dataset demonstrate that SF-HGNN achieves 82.94% ACC and 82.97% F1 in the SD experiment, and 68.85% ACC and 68.79% F1 in the SI experiment, outperforming all existing baselines. Ablation experiments verify the contribution of each module, with the removal of SF-AHGC producing the most pronounced performance degradation, confirming the central role of spatial-frequency high-order relationship modeling in enhancing emotion recognition performance. Furthermore, the frequency band boundary, hypergraph association matrix, and t-SNE visualization results jointly indicate that SF-HGNN can adaptively learn individualized frequency features, capture neurophysiologically meaningful spatial-frequency coupling patterns, and enhance the emotion discriminability of the learned representations.

Despite the above results, this study still has some limitations. First, the experiments were conducted on a single public dataset with a relatively limited sample size, which constrains the generalizability of the results to a certain extent. Second, the emotion recognition task involves only four emotion categories, and the model performance was not validated under cross-dataset conditions. In addition, the ablation experiments show that the performance gain of the DyFreBD module is relatively small, and its role mainly lies in providing better frequency band partitions for the downstream modules.

Future work will validate and extend the proposed framework on larger-scale datasets to improve the generalizability of the conclusions. Meanwhile, we will investigate cross-subject and cross-dataset domain generalization methods to enhance the robustness of the model in real-world scenarios. Furthermore, we will explore more interpretable hypergraph modeling strategies.

## Figures and Tables

**Table 1:** Hyperparameter settings of SF-HGNN.

Parameter	Value
EEG frequency bands and initial boundaries	5 bands: 0–4, 4–8, 8–14, 14–30, 30–45 Hz
fNIRS frequency bands and initial boundaries	2 bands: 0.01–0.1, 0.1–0.2 Hz
Transition-band temperature τ	EEG: 0.5; fNIRS: 0.05
Kernel sizes of the Multi-scale Temporal Convolution	EEG: 9/15/25; fNIRS: 3/5/7
Hidden dimension	32
Number of hypergraph convolution layers	3
Optimizer/learning rate/weight decay	AdamW/5 × 10^−4^/1 × 10^−4^
Label smoothing	0.1
Random seed	42

**Table 2:** Average performance of subject-dependent experiments on the ENTER dataset.

Method	Params	ACC (%)	STD (%)	F1 (%)
SVM [11]	-	51.13	6.09	50.88
TCN [37]	0.18 M	61.40	4.01	61.43
EF-Net [38]	0.26 M	64.31	5.31	64.22
EEGNet [21]	0.23 M	69.96	4.28	69.45
EFMLNet [39]	0.41 M	74.82	8.72	74.48
FGANet [4]	0.39 M	73.24	5.29	71.18
ST-DGAT [28]	1.22 M	74.48	3.79	74.51
E-FNet [40]	0.94 M	76.39	7.95	76.35
Conformer [22]	1.46 M	80.66	1.11	80.68
STA-Net [41]	1.57 M	81.17	8.82	81.15
SF-HGNN	1.29 M	82.94	3.28	82.97

**Table 3:** Average performance of subject-independent experiments on the ENTER dataset.

Method	ACC (%)	STD (%)	F1 (%)
SVM [11]	42.75	10.47	42.12
TCN [37]	47.89	6.70	47.33
EF-Net [38]	49.58	8.96	44.37
EEGNet [21]	48.91	9.70	47.79
EFMLNet [39]	48.64	9.13	47.50
FGANet [4]	60.83	8.47	60.51
ST-DGAT [28]	58.12	4.63	57.33
E-FNet [40]	51.57	7.75	51.14
Conformer [22]	66.31	5.01	65.35
STA-Net [41]	67.26	7.22	67.25
SF-HGNN	68.85	8.53	68.79

**Table 4:** Subject-dependent and subject-independent ablation experiment results on the ENTER dataset.

Method	SD			SI		
	ACC (%)	STD (%)	F1 (%)	ACC (%)	STD (%)	F1 (%)
SF-HGNN	82.94	3.28	82.97	68.85	8.53	68.79
SF-HGNN (reversed CMAF)	81.91	4.06	81.88	68.19	7.63	68.16
w/o DyFreBD	82.76	4.61	82.71	68.73	9.51	68.68
w/o MTC	81.87	3.57	81.53	68.10	9.42	68.05
w/o SF-AHGC	80.68	4.99	80.14	66.94	9.15	66.87
w/o CMAF	81.55	6.17	81.06	67.88	7.78	67.83

## Data Availability

If you are interested in the dataset and want to use it, please download and fill out the license agreement (https://gitee.com/tycgj/enter) (accessed on 8 September 2026) and send it to chenguijun@tyut.edu.cn. We will send you a download link through email after a review of your application.

## Abbreviations

The following abbreviations are used in this manuscript:

ACC	Accuracy
BN	Batch Normalization
CMAF	Cross-Modal Attention Fusion
CNN	Convolutional Neural Network
DPF	Differential Pathlength Factor
DyFreBD	Dynamic Frequency Band Decomposition
EEG	Electroencephalography
ELU	Exponential Linear Unit
F1	Macro F1-Score
fNIRS	Functional Near-Infrared Spectroscopy
GAT	Graph Attention Network
GCN	Graph Convolutional Network
GELU	Gaussian Error Linear Unit
GNN	Graph Neural Network
HbO	Oxygenated Hemoglobin
HbR	Deoxygenated Hemoglobin
HGNN	Hypergraph Neural Network
IRFFT	Inverse Real Fast Fourier Transform
LOSO	Leave-One-Subject-Out
MTC	Multi-scale Temporal Convolution
ReLU	Rectified Linear Unit
RFFT	Real Fast Fourier Transform
SD	Subject-Dependent
SF-AHGC	Spatial-Frequency Adaptive Hypergraph Convolution
SF-HGNN	Spatial-Frequency Hypergraph Neural Network
SI	Subject-Independent
STD	Standard Deviation
SVM	Support Vector Machine
TCN	Temporal Convolutional Network
t-SNE	t-distributed Stochastic Neighbor Embedding
